# Differences in Next-Day Adverse Effects and Impact on Mood of an Evening of Heavy Alcohol Consumption between Hangover-Sensitive Drinkers and Hangover-Resistant Drinkers

**DOI:** 10.3390/jcm12062090

**Published:** 2023-03-07

**Authors:** Marlou Mackus, Aurora J. A. E. van de Loo, Renier H. P. van Neer, Sterre A. Vermeulen, Chantal Terpstra, Karel A. Brookhuis, Johan Garssen, Andrew Scholey, Joris C. Verster

**Affiliations:** 1 Division of Pharmacology, Utrecht Institute for Pharmaceutical Sciences, Utrecht University, 3584CG Utrecht, The Netherlands; 2 Centre for Human Psychopharmacology, Swinburne University, Melbourne, VIC 3122, Australia; 3 Faculty of Behavioral and Social Sciences, Groningen University, 9700AB Groningen, The Netherlands; 4 Global Centre of Excellence Immunology, Nutricia Danone Research, 3584CT Utrecht, The Netherlands; 5 Nutrition Dietetics and Food, School of Clinical Sciences, Monash University, Melbourne, VIC 3168, Australia

**Keywords:** alcohol, hangover, adverse effects, mood, sleepiness, symptoms, hangover resistance

## Abstract

The combination of negative mental and physical symptoms which can be experienced after a single episode of alcohol consumption, starting when blood alcohol concentration (BAC) approaches zero, are collectively referred to as the alcohol hangover. Previous research revealed that 10 to 20% of drinkers claim not to experience next-day hangovers. Past studies were usually limited to single timepoint assessments. The aim of the current semi-naturalistic study was to compare the next-day effects of an evening of alcohol consumption of self-reported hangover-resistant drinkers (n = 14) with those of a group of self-reported hangover-sensitive drinkers (n = 15) at hourly timepoint throughout the day (09:30 until 15:30). Assessments of 23 hangover symptoms, mood (Profiles of Mood States-Short Form), and daytime sleepiness (Karolinska Sleepiness Scale) were made hourly after both an alcohol day and an alcohol-free control day. Additional morning assessments were made for mood (State-Trait Anxiety Inventory-Y, Beck’s Depression Inventory-II), risk-taking behavior (RT-18), past night sleep (Groningen Sleep Quality Scale), alcohol consumption, and activities during the test days. No significant differences were found regarding the amount of alcohol consumed and the total sleep time of the two groups. The hangover-sensitive group reported having a hangover as well as the presence of a variety of hangover-related symptoms, which were most severe in the morning and then gradually decreased during the day. The most frequently reported and most severe symptoms were sleepiness and fatigue, concentration problems, and headache. In contrast, the hangover-resistant group reported the absence of a hangover and the presence and severity of next-day symptoms did not significantly differ from the control day, except for increased fatigue and reduced vigor. The next-day effects on sleepiness-related complaints and vigor were significantly more pronounced among hangover-sensitive drinkers compared to hangover-resistant drinkers. In conclusion, contrary to hangover-resistant drinkers, hangover-sensitive drinkers report a variety of hangover symptoms that gradually ease during the day, but are still present in the afternoon.

## 1. Introduction

The alcohol hangover refers to the combination of negative mental and physical symptoms which can be experienced after a single episode of alcohol consumption, starting when blood alcohol concentration (BAC) approaches zero [1]. Among the most frequently reported adverse effects are being tired, sleepiness, concentration problems, and headache [2,3,4]. There is increasing research into the pathology of alcohol hangovers [5,6,7]. Current theories suggest that the alcohol hangover comprises an inflammatory response to alcohol consumption and that this immune response, in combination with variations in alcohol metabolism, determines the presence and severity of next-day hangovers [8,9,10]. 

In the past, it was thought that the next-day adverse effects would only be present after consuming significant amounts of alcohol, exceeding a BAC of 0.11% [11]. However, recent research revealed that hangovers can occur after consuming any amount of alcohol, depending on the individual drinker [12]. Other studies showed that around 10 to 20% of drinkers claim not to have a hangover, despite consuming large quantities of alcohol [13,14,15]. These hangover-resistant drinkers do not report the common next-day hangover symptoms, except for fatigue and sleepiness-related symptoms, which can probably be related to the sleep loss associated with an evening out instead of the alcohol consumption itself. There is little research comparing the next-day effects reported by hangover-sensitive drinkers and hangover-resistant drinkers. A naturalistic study by Hogewoning et al. [16] compared hangover-resistant drinkers with hangover-sensitive drinkers. No profound hangover effects were found for the hangover-resistant group. Compared to the control day, following the alcohol day small but significant increases were found for sleepiness (1.2 to 2.1, respectively), tiredness (1.2 to 2.5, respectively), concentration problems (0.3 to 1.1, respectively), clumsiness (0.1 to 0.7, respectively), and thirst (1.1 to 2.5, respectively); they also reported poorer sleep quality, but no effects were found for mood, including anxiety and depression. Taken together, these findings suggest that in hangover-resistant drinkers the effects of an evening of alcohol consumption are limited to poorer sleep and mild next-day fatigue-related symptoms. In contrast, the hangover-sensitive group reported a hangover, a significant increase in various hangover symptoms, and a reduction in mood the day after alcohol consumption. However, the observations were limited to a single assessment in the morning (09:30) on the alcohol and control day. Research revealed, however, that hangover severity may vary during the day and individual differences in severity patterns over time have been identified [17]. Therefore, the aim of the current study was to more comprehensively investigate the next-day effects of an evening of alcohol consumption, compared to an alcohol-free (control) evening. Assessments were made hourly throughout the day, from 09:30 to 15:30, on both the alcohol day and the control day. The study had a naturalistic design [18] in which participants could consume alcohol freely (type and amount of consumed alcohol, and start and stop times of drinking were chosen by the participants), and they engaged in their usual activities (e.g., visit a pub, dancing, or staying at home). The naturalistic design was chosen as, in contrast to a controlled clinical trial, this design most closely mimics a real-life drinking experience [18]. Overall hangover severity and the severities of 22 individual hangover symptoms were assessed hourly. Also completed every hour were the Karolinska Sleepiness Scale (KSS) [19], and the Profiles of Mood Scale-Short Form (POMS-SF) [20]. Additional information on sleep and mood on both test days was collected with the Groningen Sleep Quality Scale (GSQS) [21], State Trait Anxiety Inventory-Y (STAI-Y) [22], Beck’s Depression Inventory-II (BDI-II) [23,24,25], and the 18-item risk-taking questionnaire (RT-18) [26], respectively. It was hypothesized that the hangover-sensitive group would report most of the hangover symptoms, most pronounced in the morning assessments and then gradually decreasing throughout the day. In contrast, it was hypothesized that next-day effects reported by the hangover-resistant group, if any, would be limited to sleepiness-related symptoms. 

## 2. Methods

A semi-naturalistic study was conducted [18], comprising a training day and two test days. The evening and night of the test days comprised a naturalistic study design. On the control day, no alcohol was consumed in the evening. On the alcohol day, participants consumed alcohol (type and quantity of alcohol of choice). In order to closely mimic a real-life drinking experience, there were no restrictions on venue, activity, or behaviors on the test days. The next morning (09:00), participants came to Utrecht University where the assessments were conducted, applying a strictly controlled experimental study design, including standardized validated assessments and study procedures at pre-set times. The study was approved by the University of Groningen Psychology Ethics Committee (approval number: ppo-015-002, approval date: 3 September 2015). Written informed consent was obtained from all participants before the start of the study. 

### 2.1. Screening Assessments and Eligibility of Participants

Participants were included in the study if they had Dutch nationality, were between 18 and 30 years old, and consumed alcohol. Participants had to be healthy (i.e., no physical or mental disease), non-smoking, and not using illicit or medicinal drugs (except contraception). Health status was verified by the study physician, and sex, age, weight, and height were recorded. At screening, and on each test day, the absence of illicit drug use (including amphetamines (including 3,4-Methylenedioxymethamphetamine, MDMA), barbiturates, cannabinoids, benzodiazepines, cocaine, and opiates) was verified via a urine drug screen (AlfaScientic Designs Inc., Poway, CA, USA) and female participants completed a urine β-human chorionic gonadotropin (HCG) pregnancy test. None of the participants tested positive on these tests. A breath alcohol test was performed using an Alcotest 7410 Breath Alcoholmeter (Dräger, Hoogvliet, The Netherlands) to verify the absence of alcohol consumption on the control day and possible residual alcohol on the alcohol day. On the test days, participants were not allowed to take any treatments to prevent or relieve hangover symptoms or medicinal drugs that may potentially reduce hangover symptoms such as painkillers (e.g., acetaminophen or aspirin). 

At screening, potential participants were thoroughly interviewed about their drinking behaviors and history of presence or absence of alcohol hangovers. The purpose of the study was to compare hangover-resistant drinkers with hangover-sensitive drinkers. Therefore, participants were included that reported consuming considerable amounts of alcohol on a regular drinking occasion. Although recent research revealed that hangovers can occur after consuming any amount of alcohol [12], for the current study participants were included only if—according to their dinking history interview—they consumed alcohol on a regular drinking occasion that resulted in a corresponding peak BAC of at least 0.08%. The peak BAC was determined by using a modified Widmark formula [27,28], which takes drinking time and the amount of alcohol consumed into account and controls for sex and body weight. When participants reported the absence of an alcohol hangover the day after such a drinking occasion, they were allocated to the hangover-resistant group and when they reported a hangover, they were allocated to the hangover-sensitive group.

Baseline sleep was assessed with the SLEEP-50 questionnaire [29]. The SLEEP-50 comprises 50 items that form nine subscales. The subscales assess sleep apnea, insomnia, narcolepsy, restless legs/periodic leg movement disorder, circadian rhythm sleep disorder, sleepwalking, nightmares, factors influencing sleep, and the impact of sleep complaints on daily functioning. Each item is scored on a 4-point scale: 1 (not at all), 2 (somewhat), 3 (rather much), and 4 (very much). Higher scores on the scales indicate poorer sleep or poorer daytime functioning. Cut-off values for a positive screen for sleep disorders include ≥15 on the sleep apnea scale (sensitivity = 85%, specificity = 88%), ≥19 on the insomnia scale (sensitivity = 71%, specificity = 75%), ≥7 on the restless legs/periodic leg movement disorder scale (sensitivity = 83%, specificity = 72%), and ≥8 on the circadian rhythm sleep disorder scale (sensitivity = 83%, specificity = 69%). In addition to the SLEEP-50, ‘common’ average total sleep time was recorded, and usual sleep quality was rated on a scale ranging from 0 (very poor) to 10 (very good). 

### 2.2. Procedures on Test Days

Visits were scheduled approximately one week apart. However, participants could (last-minute) decide not to drink alcohol on the planned alcohol test day. In that case, the alcohol test day was postponed. All participants completed an alcohol test day and a control test day. On both test days, participants arrived at Utrecht University at 09:00. They received a standardized breakfast (a currant bun and a glass of milk or water) and a similar lunch at midday. They were instructed not to consume caffeinated beverages on test days. Assessments were made throughout the days, at hourly intervals, starting at 09:30 until 15:30.

### 2.3. Single-Time Assessments at Arrival

Participants reported the amount of alcohol they consumed the previous evening, and the start and stop time of alcohol consumption. Guidance was provided on how to report the quantity of alcohol consumed and how to convert drink sizes or bottles to standard units (in The Netherlands, standard units of alcoholic drinks each contain 10 g of alcohol, independent of the type of alcoholic beverage). Bodyweight was measured on each test day, and estimated BAC was computed.

Activities and visited venues during the drinking session and the control evening were recorded. These varied between participants and were categorized as (a) being at home (not active), (b) in the pub (not active), or (c) party (dancing) or sports (active). 

Anxiety on the alcohol and control day was assessed with the State-Trait Anxiety Inventory-Y (STAI-Y) [22]. The STAI-Y measures both state (momentary) anxiety (STAI-S, 20 items) and trait (baseline) anxiety (STAI-T, 20 items). Items are scored on a 4-point Likert scale (range 1 to 4). Overall STAI-S and STAI-T scores range from 20 to 80, with higher scores indicating more anxiety. Scores can be classified as “no or low anxiety” (score range 20–37), “moderate anxiety” (score range 38–44), or “high anxiety” (score range 45–80) [22].

Depression was assessed on the alcohol and control day with the Beck’s Depression Inventory-II (BDI-II) [23,24,25]. The BDI-II comprises 21 questions which can be scored on a 4-point scale ranging from 0 to 3 based on the severity of each item. The sum of items is computed to represent the overall depression score. Lower scores indicate less severe depressive symptoms. Cut-off ranges for the BDI-II include 29–63 for severe depression, 20–28 for moderate depression, 14–19 for mild depression, and 0–13 for minimal or no depression [25]. 

On both test days, risk-taking behavior was assessed with the 18-item risk-taking (RT-18) questionnaire [26]. The items have a yes/no answering format (scored 0 or 1, including reversed-scored items). The overall RT-18 score ranges from 0 (no risk-taking) to 18 (extreme risk-taking). The RT-18 has two subscales labeled factor 1 (risk-taking) and factor 2 (risk assessment), with scores ranging from 0 to 9. Higher scores on these scales indicate being engaged in more risk-taking behavior (factor 1) and a poorer assessment of risk (factor 2). 

On both test days, the quality of the previous night’s sleep was assessed with the Groningen Sleep Quality Scale (GSQS) [21]. Participants answered 14 items on sleep quality (yes/no format). The overall GSQS score ranges from 0 to 14, with higher scores indicating poorer sleep quality. Also recorded was the time to fall asleep and wake up (which enabled the calculation of total sleep time), and the number of nightly awakenings.

### 2.4. Hourly Assessments

Hourly assessments started at 09:30 and ended at 15:30. Overall hangover severity was assessed using a single-item scale ranging from 0 (absent) to 10 (extreme) [30]. Using the same 11-point scale, the severity of 23 individual symptoms that are frequently related to the hangover state were assessed. The symptoms were a combination of the symptoms of three existing hangover scales [31,32,33] and included fatigue, sleepiness, apathy, concentration problems, headache, nausea, regret, heart pounding, heart racing, vomiting, shivering, clumsiness, weakness, dizziness, sweating, stomach pain, confusion, sensitivity to light, sensitivity to sound, thirst, anxiety, depression, and reduced appetite.

The Karolinska Sleepiness Scale (KSS) was completed to rate their current level of sleepiness [19]. Participants could choose one of nine statements, ranging from ‘extremely alert’ (score of 1) to ‘extremely sleepy, fighting sleep’ (score of 9). Mood was assessed using a Dutch version of the Profiles of Mood States-Short Form (POMS-SF) [20]. The Dutch POMS-SF consists of 32 items which are scored on a 5-point Likert scale. Scores range from 0 (not at all) to 4 (extremely). The Dutch POMS-SF has five subscales assessing tension, depression, anger, vigor, and fatigue. 

### 2.5. Statistical Analysis

Statistical analyses were performed using IBM Statistical Package for the Social Sciences (SPSS), version 29. The analyses compared the outcomes of the hangover-sensitive group and the hangover-resistant group. For demographics and baseline assessments, the comparisons were made with the non-parametric independent-samples Mann–Whitney U test. Percentual data were compared with the Chi-square test (Fischer exact test, 2-sided). Differences were considered significant if *p* < 0.05. For single timepoint assessments, comparisons within groups (alcohol day versus control day) were made with the Related-Samples Wilcoxon Signed Ranks Test. Differences were considered significant if *p* < 0.05. For multiple timepoint assessments, the groups were compared with the non-parametric Independent-Samples Kruskal-Wallis test. Differences (at the same timepoint) between the hangover-resistant group and the hangover-sensitive group, after Bonferroni’s correction for multiple comparisons, were considered significant if *p* < 0.0071. Data from the control day and the hangover day were compared within groups with the non-parametric Related-samples Friedman’s Two-Way Analysis of Variance by Ranks Test. Differences (at the same timepoint) between the control day and the alcohol day, after Bonferroni’s correction for multiple comparisons, were considered significant if *p* < 0.0071.

## 3. Results

N = 29 healthy volunteers participated in the study. Of them, 14 were allocated to the hangover-resistant group and 15 to the hangover-sensitive group. 

### 3.1. Demographics and Baseline Sleep

Demographics and baseline sleep assessments are summarized in Table 1. The groups did not significantly differ in the assessed demographics or sleep outcomes. 

### 3.2. Activity on the Test Days

Participants were free to engage in their usual activities on both test days. A summary of their activities is given in Table 2. On the control day, the majority of participants stayed at home (24 of 29 participants), whereas on the alcohol day about half of the participants went to a pub or party (15 of 29 participants). However, no significant differences were found between the alcohol day and the control day (*p* = 0.059 for both groups). Moreover, no significant differences in activity were found between the hangover-resistant group and the hangover-sensitive group on the control day (*p* = 0.481) or the alcohol day (*p* = 0.401).

### 3.3. Estimated Alcohol Consumption

As instructed, participants consumed no alcohol on the control day. Alcohol consumption outcomes for the alcohol day are summarized in Table 3. These were estimated by the participants the morning following drinking. The hangover-resistant group and hangover-sensitive group did not significantly differ in the amount of alcohol consumed or the estimated BAC on the alcohol day. The latter is an important observation, as it warrants a fair comparison of other variables (e.g., the presence and severity of adverse effects) of both groups.

### 3.4. Sleep on Test Days

Sleep quality on the alcohol and control day was assessed with the Groningen Sleep Quality Scale (GSQS). The results are summarized in Table 4. In both groups, on the alcohol day time to bed (TTB), time lights out (TLO), and time falling asleep (TFA) were significantly later compared to the control day. As no significant differences were reported for wake-up time (WUT) on both test days, the corresponding total sleep time (TST) of both groups was significantly shorter on the alcohol day (~75 min shorter in both groups compared to the control day). Thus, for both groups drinking time goes at the expense of total sleep time. Whereas sleep quality was not significantly affected by alcohol consumption in the hangover-resistant group, hangover-sensitive drinkers had a significantly poorer sleep quality score on the alcohol day compared to the control day. On the control day, sleep quality did not differ between the groups. On the alcohol day, sleep quality was significantly poorer in the hangover-sensitive group than in the hangover-resistant group. 

### 3.5. Mood Assessments (in the Morning after Arrival)

Directly after arrival in the morning, several mood assessments were made. The outcomes of these assessments on anxiety, depression, and risk-taking behavior are summarized in Table 5. Risk-taking did not differ between the groups or between the alcohol and control days. On the alcohol day, and increase in STAI-S was found for the hangover-sensitive group.

On average, the anxiety scores on the STAI were below the cut-off of 37, corresponding to no or low anxiety [22]. Moreover, average depression scores on the BDI-II were below 9, indicating no or minimal depression [25]. The clinical relevance of the observed differences between the hangover-resistant group and the hangover-sensitive group is therefore unclear. In the hangover-resistant group, no significant differences in anxiety and depression were found between the control day and the alcohol day. The hangover-sensitive group had a small but significant increase in STAI-State score on the alcohol day. No significant effects on risk-taking behavior were found.

### 3.6. Overall Hangover Severity

**Figure 1:**
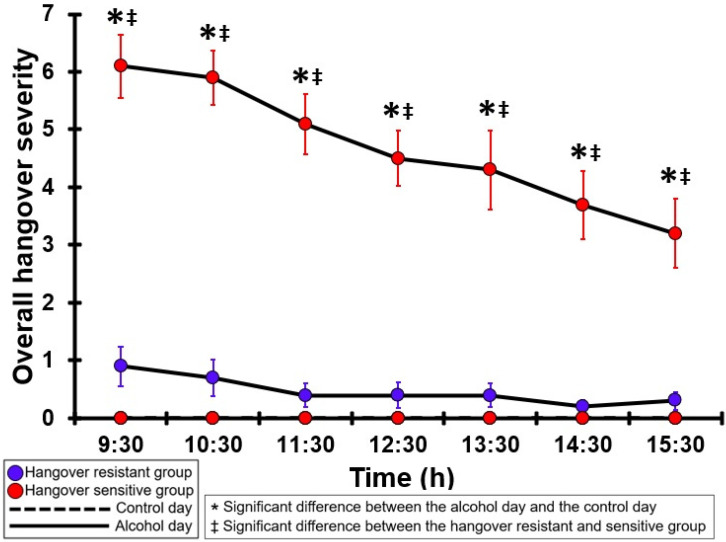
Overall hangover severity. Mean and standard error (SE) are shown. Significant differences (at the same timepoint) between the control day and the alcohol day (*p* < 0.0071, after Bonferroni’s correction for multiple comparisons) are indicated by *. Significant differences (at the same timepoint) between the hangover-resistant group and the hangover-sensitive group (*p* < 0.0071, after Bonferroni’s correction for multiple comparisons) are indicated by ‡.

Table A1 and Figure 1 summarize the overall hangover severity ratings of the hangover-resistant group and the hangover-sensitive group. For the hangover-resistant group, no significant differences were found between the alcohol and control day. The average ratings on both test days remained below 1 (out of 10). For the hangover-sensitive group, the overall hangover rating at 09:30 on the alcohol day was 6.1 and then gradually decreased during the day. For all timepoints hangover severity ratings on the alcohol day were significantly higher than those on the control day. On the control day, overall hangover severity ratings were zero in both groups and thus did not significantly differ between the groups. On the alcohol day, for all timepoints, overall hangover severity ratings of the hangover-sensitive group were significantly higher than those of the hangover-resistant group. 

### 3.7. Fatigue

**Figure 2:**
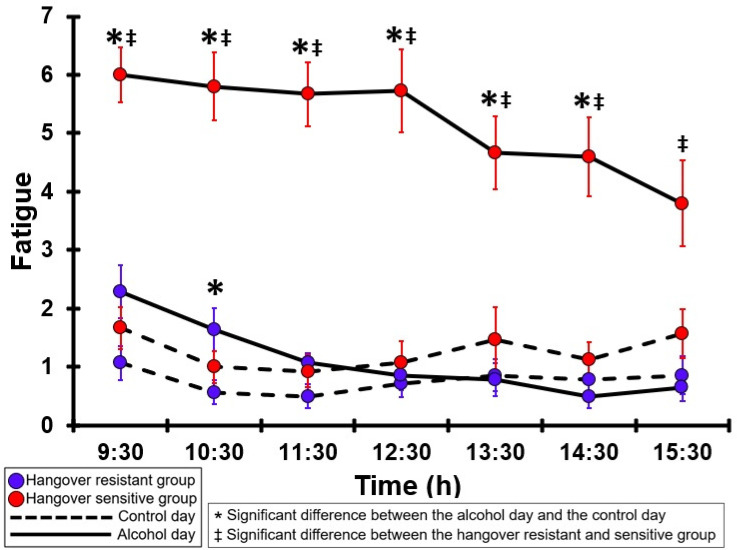
Fatigue. Mean and standard error (SE) are shown. Significant differences (at the same timepoint) between the control day and the alcohol day (*p* < 0.0071, after Bonferroni’s correction for multiple comparisons) are indicated by *. Significant differences (at the same timepoint) between the hangover-resistant group and the hangover-sensitive group (*p* < 0.0071, after Bonferroni’s correction for multiple comparisons) are indicated by ‡.

Table A2 and Figure 2 summarize the fatigue ratings of the hangover-resistant group and the hangover-sensitive group. For the hangover-resistant group, no significant differences were found between the alcohol and control days, except for a small significant increase at 10:30. The average ratings on both test days remained below 3 (out of 10). For the hangover-sensitive group, the fatigue rating at 09:30 on the alcohol day was 6.0 and then gradually decreased during the day. For all timepoints except 15:30, fatigue ratings on the alcohol day were significantly higher than those on the control day. On the control day, fatigue ratings did not significantly differ between the hangover-resistant group and the hangover-sensitive group. On the alcohol day, for all timepoints fatigue ratings of the hangover-sensitive group were significantly higher than those of the hangover-resistant group. 

### 3.8. Sleepiness

**Figure 3:**
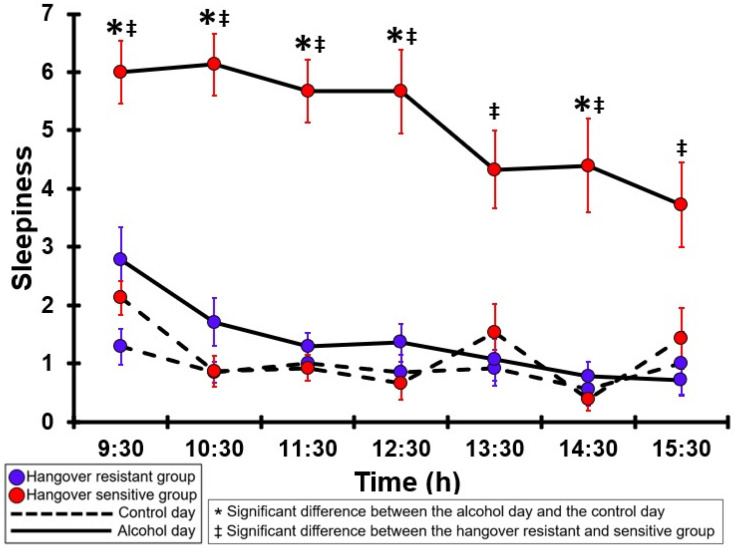
Sleepiness. Mean and standard error (SE) are shown. Significant differences (at the same timepoint) between the control day and the alcohol day (*p* < 0.0071, after Bonferroni’s correction for multiple comparisons) are indicated by *. Significant differences (at the same timepoint) between the hangover-resistant group and the hangover-sensitive group (*p* < 0.0071, after Bonferroni’s correction for multiple comparisons) are indicated by ‡.

Table A3 and Figure 3 summarize the sleepiness ratings of the hangover-resistant group and the hangover-sensitive group. For the hangover-resistant group, no significant differences were found between the alcohol and control days. The average ratings on both test days remained below 3 (out of 10). For the hangover-sensitive group, the sleepiness rating at 09:30 on the alcohol day was 6.0 and then gradually decreased during the day. For all timepoints except 13:30 and 15:30 sleepiness ratings on the alcohol day were significantly higher than those on the control day. On the control day, sleepiness ratings did not significantly differ between the hangover-resistant group and the hangover-sensitive group. On the alcohol day, for all timepoints sleepiness ratings of the hangover-sensitive group were significantly higher than those of the hangover-resistant group. 

### 3.9. Apathy

**Figure 4:**
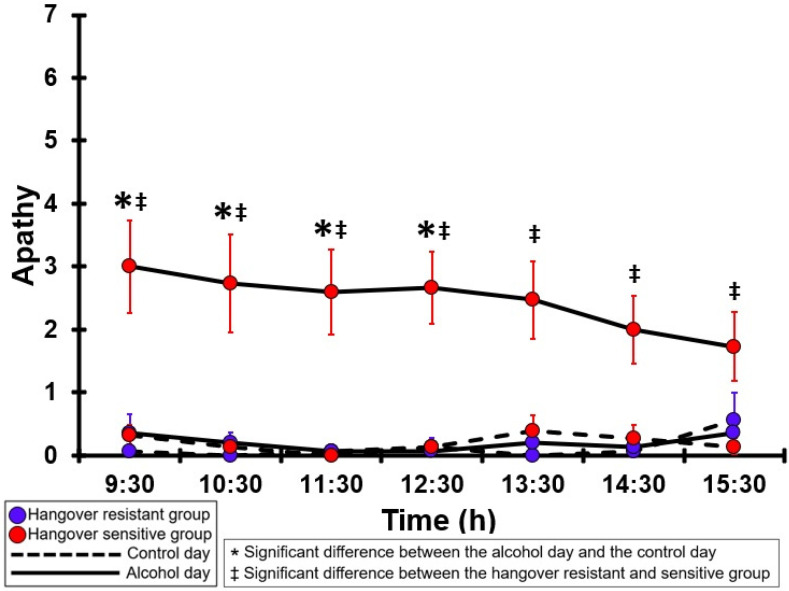
Apathy. Mean and standard error (SE) are shown. Significant differences (at the same timepoint) between the control day and the alcohol day (*p* < 0.0071, after Bonferroni’s correction for multiple comparisons) are indicated by *. Significant differences (at the same timepoint) between the hangover-resistant group and the hangover-sensitive group (*p* < 0.0071, after Bonferroni’s correction for multiple comparisons) are indicated by ‡.

Table A4 and Figure 4 summarize the apathy ratings of the hangover-resistant group and the hangover-sensitive group. For the hangover-resistant group, no significant differences were found between the alcohol and control days. The average ratings on both test days remained below 1 (out of 10). For the hangover-sensitive group, the apathy rating at 09:30 on the alcohol day was 3.0 and then gradually decreased during the day. For the timepoints 09:30 to 12:30, apathy ratings on the alcohol day were significantly higher than those on the control day. On the control day, apathy ratings did not significantly differ between the hangover-resistant group and the hangover-sensitive group. On the alcohol day, for all timepoints apathy ratings of the hangover-sensitive group were significantly higher than those of the hangover-resistant group.

### 3.10. Concentration Problems

**Figure 5:**
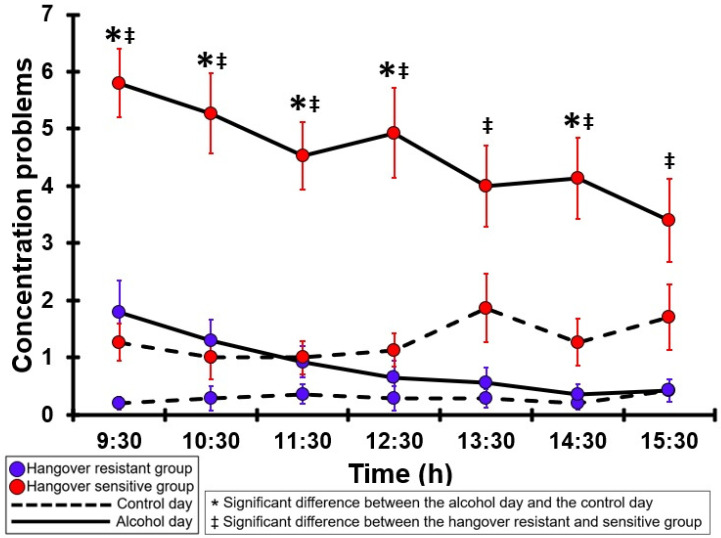
Concentration problems. Mean and standard error (SE) are shown. Significant differences (at the same timepoint) between the control day and the alcohol day (*p* < 0.0071, after Bonferroni’s correction for multiple comparisons) are indicated by *. Significant differences (at the same timepoint) between the hangover-resistant group and the hangover-sensitive group (*p* < 0.0071, after Bonferroni’s correction for multiple comparisons) are indicated by ‡.

Table A5 and Figure 5 summarize the concentration problems ratings of the hangover-resistant group and the hangover-sensitive group. For the hangover-resistant group, no significant differences were found between the alcohol and control days. The average ratings on both test days remained below 2 (out of 10). For the hangover-sensitive group, the concentration problems rating at 09:30 on the alcohol day was 5.8 and then gradually decreased during the day. For the timepoints 09:30 to 12:30, concentration problems ratings on the alcohol day were significantly higher than those on the control day. On the control day, concentration problems ratings did not significantly differ between the hangover-resistant group and the hangover-sensitive group. On the alcohol day, for all timepoints concentration problems ratings of the hangover-sensitive group were significantly higher than those of the hangover-resistant group. 

### 3.11. Weakness

**Figure 6:**
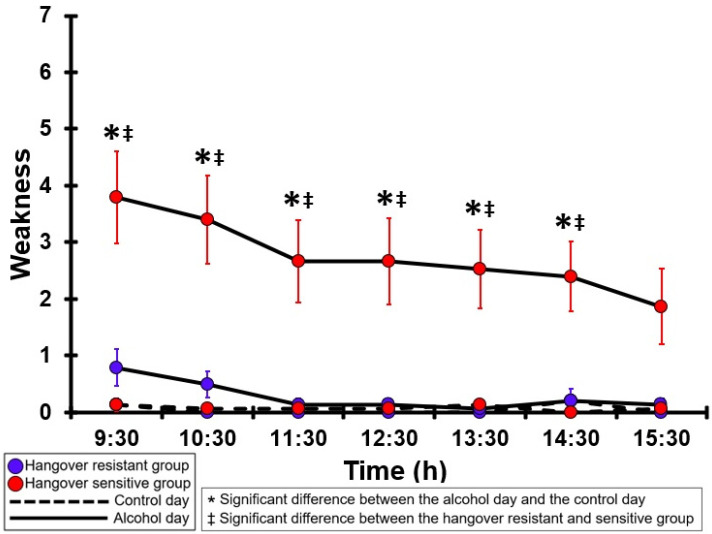
Weakness. Mean and standard error (SE) are shown. Significant differences (at the same timepoint) between the control day and the alcohol day (*p* < 0.0071, after Bonferroni’s correction for multiple comparisons) are indicated by *. Significant differences (at the same timepoint) between the hangover-resistant group and the hangover-sensitive group (*p* < 0.0071, after Bonferroni’s correction for multiple comparisons) are indicated by ‡.

Table A6 and Figure 6 summarize the weakness ratings of the hangover-resistant group and the hangover-sensitive group. For the hangover-resistant group, no significant differences were found between the alcohol and control days. The average ratings on both test days remained below 1 (out of 10). For the hangover-sensitive group, the weakness rating at 09:30 on the alcohol day was 3.8 and then gradually decreased during the day. For all timepoints except 15:30 weakness ratings on the alcohol day were significantly higher than those on the control day. On the control day, weakness ratings did not significantly differ between the hangover-resistant group and the hangover-sensitive group. On the alcohol day, for all timepoints except 15:30 weakness ratings of the hangover-sensitive group were significantly higher than those of the hangover-resistant group. 

### 3.12. Clumsiness

**Figure 7:**
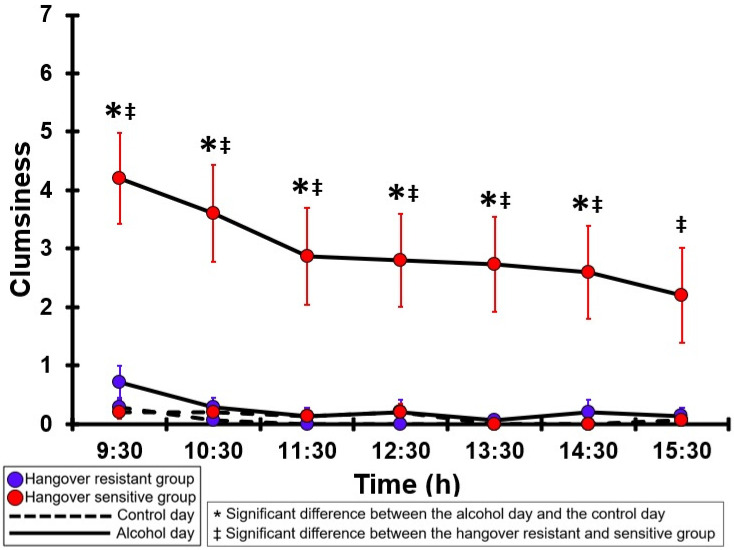
Clumsiness. Mean and standard error (SE) are shown. Significant differences (at the same timepoint) between the control day and the alcohol day (*p* < 0.0071, after Bonferroni’s correction for multiple comparisons) are indicated by *. Significant differences (at the same timepoint) between the hangover-resistant group and the hangover-sensitive group (*p* < 0.0071, after Bonferroni’s correction for multiple comparisons) are indicated by ‡.

Table A7 and Figure 7 summarize the clumsiness ratings of the hangover-resistant group and the hangover-sensitive group. For the hangover-resistant group, no significant differences were found between the alcohol and control days. The average ratings on both test days remained below 1 (out of 10). For the hangover-sensitive group, the clumsiness rating at 09:30 on the alcohol day was 4.2 and then gradually decreased during the day. For all timepoints except 15:30, clumsiness ratings on the alcohol day were significantly higher than those on the control day. On the control day, clumsiness ratings did not significantly differ between the hangover-resistant group and the hangover-sensitive group. On the alcohol day, for all timepoints clumsiness ratings of the hangover-sensitive group were significantly higher than those of the hangover-resistant group. 

### 3.13. Confusion

**Figure 8:**
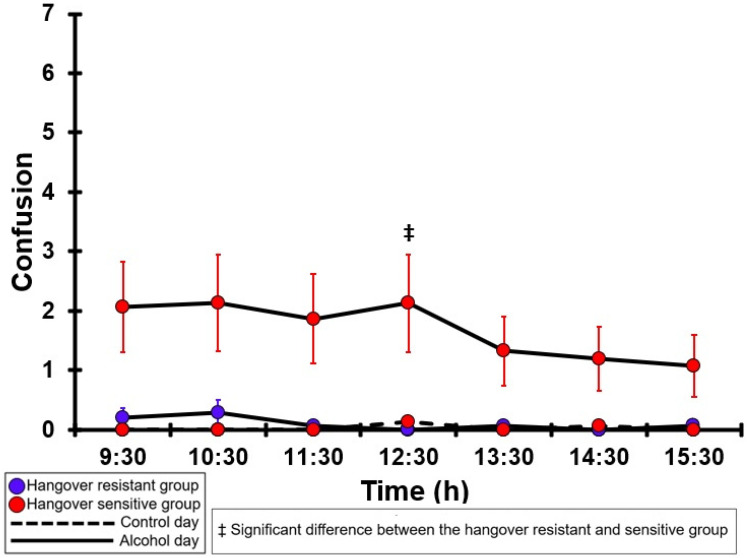
Confusion. Mean and standard error (SE) are shown. No significant differences (at the same timepoint) between the control day and the alcohol day (*p* < 0.0071, after Bonferroni’s correction for multiple comparisons) were found. Significant differences (at the same timepoint) between the hangover-resistant group and the hangover-sensitive group (*p* < 0.0071, after Bonferroni’s correction for multiple comparisons) are indicated by ‡.

Table A8 and Figure 8 summarize the confusion ratings of the hangover-resistant group and the hangover-sensitive group. For the hangover-resistant group, no significant differences were found between the alcohol and control days. The average ratings on both test days remained below 1 (out of 10). For the hangover-sensitive group, the confusion rating at 09:30 on the alcohol day was 2.1 and then gradually decreased during the day. However, no significant differences were found between the alcohol and control day. On both the control and alcohol day, no significant differences in confusion ratings were found between the hangover-resistant group and the hangover-sensitive group, except at 12:30 on the alcohol day when the confusion rating was significantly higher in the hangover-sensitive group.

### 3.14. Headache

**Figure 9:**
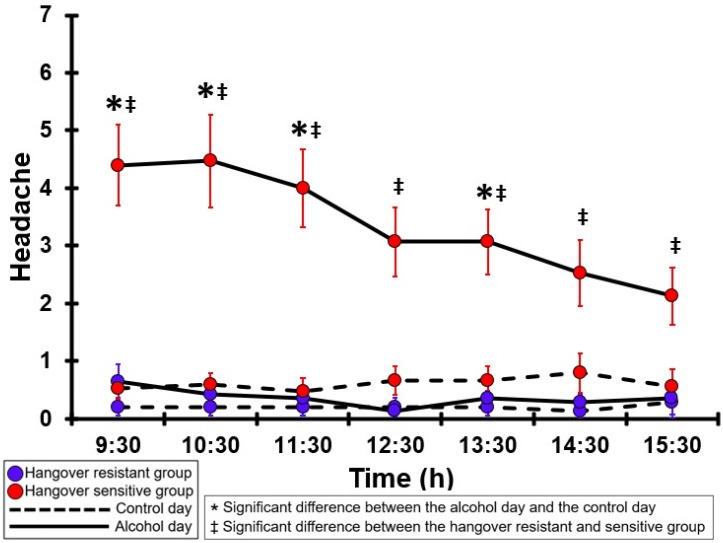
Headache. Mean and standard error (SE) are shown. Significant differences (at the same timepoint) between the control day and the alcohol day (*p* < 0.0071, after Bonferroni’s correction for multiple comparisons) are indicated by *. Significant differences (at the same timepoint) between the hangover-resistant group and the hangover-sensitive group (*p* < 0.0071, after Bonferroni’s correction for multiple comparisons) are indicated by ‡.

Table A9 and Figure 9 summarize the headache ratings of the hangover-resistant group and the hangover-sensitive group. For the hangover-resistant group, no significant differences were found between the alcohol and control days. The average ratings on both test days remained below 1 (out of 10). For the hangover-sensitive group, the headache rating at 09:30 on the alcohol day was 4.4 and then gradually decreased during the day. Significant differences were found between the alcohol and control days from 09:30 to 11:30 and at 13:30. On the control day, no significant differences in headache ratings were found between the hangover-resistant group and the hangover-sensitive group. On the alcohol day, at each timepoint headache ratings of the hangover-sensitive group were significantly higher than those of the hangover-resistant group. 

### 3.15. Stomach Pain

**Figure 10:**
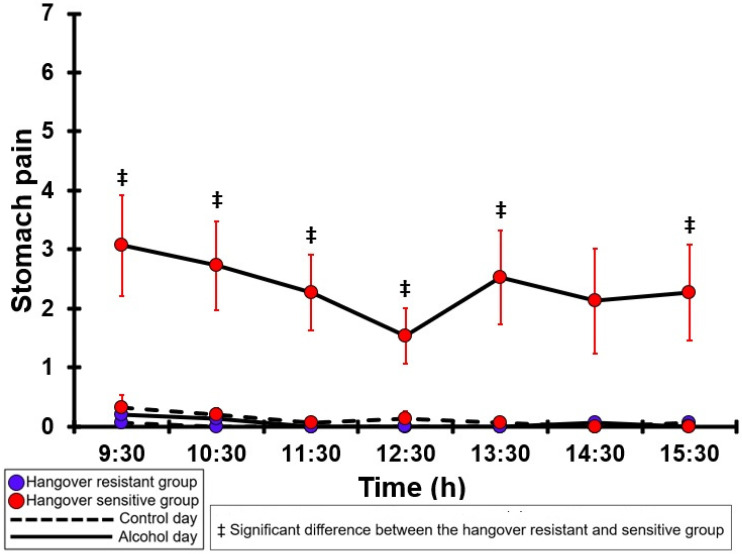
Stomach pain. Mean and standard error (SE) are shown. No significant differences (at the same timepoint) between the control day and the alcohol day (*p* < 0.0071, after Bonferroni’s correction for multiple comparisons) were found. Significant differences (at the same timepoint) between the hangover-resistant group and the hangover-sensitive group (*p* < 0.0071, after Bonferroni’s correction for multiple comparisons) are indicated by ‡.

Table A10 and Figure 10 summarize the stomach pain ratings of the hangover-resistant group and the hangover-sensitive group. For both the hangover-resistant group and the hangover-sensitive group, no significant differences were found between the alcohol and control days. On both test days, the average stomach pain ratings of the hangover-resistant group remained below 1 (out of 10). For the hangover-sensitive group, the stomach pain rating at 09:30 on the alcohol day was 3.1 and then gradually decreased during the day. On the control day, no significant differences in stomach pain ratings were found between the hangover-resistant group and the hangover-sensitive group. On the alcohol day, at each timepoint except 14:30 stomach pain ratings of the hangover-sensitive group were significantly higher than those of the hangover-resistant group. 

### 3.16. Nausea

**Figure 11:**
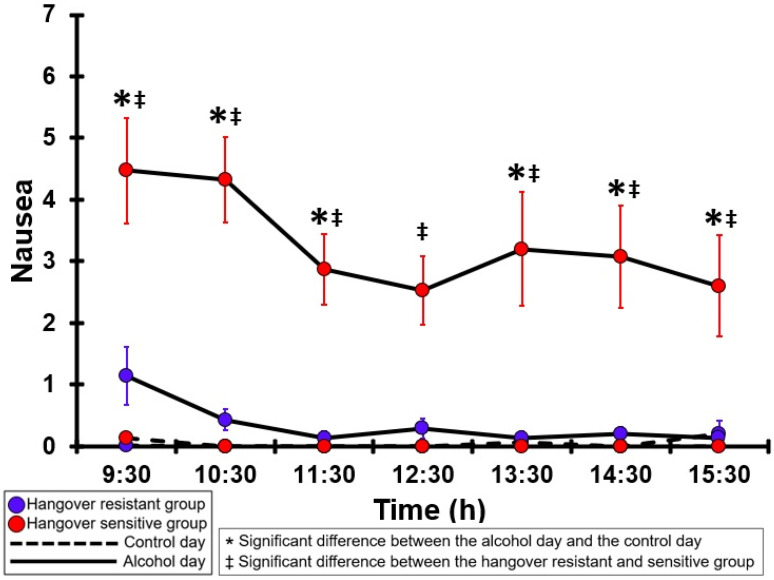
Nausea. Mean and standard error (SE) are shown. Significant differences (at the same timepoint) between the control day and the alcohol day (*p* < 0.0071, after Bonferroni’s correction for multiple comparisons) are indicated by *. Significant differences (at the same timepoint) between the hangover-resistant group and the hangover-sensitive group (*p* < 0.0071, after Bonferroni’s correction for multiple comparisons) are indicated by ‡.

Table A11 and Figure 11 summarize the nausea ratings of the hangover-resistant group and the hangover-sensitive group. For the hangover-resistant group, no significant differences were found between the alcohol and control days. On both test days, the average nausea ratings of the hangover-resistant group remained below 2 (out of 10). For the hangover-sensitive group, the nausea rating at 09:30 on the alcohol day was 4.5 and then gradually decreased during the day. Significantly higher nausea ratings on the alcohol day were found for each timepoint except at 12:30. On the control day, no significant differences in nausea ratings were found between the hangover-resistant group and the hangover-sensitive group. On the alcohol day, at each timepoint nausea ratings of the hangover-sensitive group were significantly higher than those of the hangover-resistant group. 

### 3.17. Vomiting

**Figure 12:**
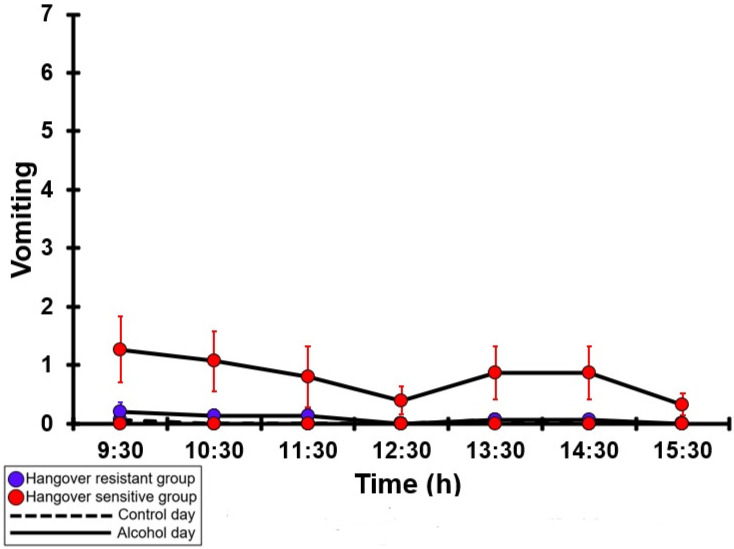
Vomiting. Mean and standard error (SE) are shown. No significant differences (at the same timepoint) between the control day and the alcohol day (*p* < 0.0071, after Bonferroni’s correction for multiple comparisons) were found. No significant differences (at the same timepoint) between the hangover-resistant group and the hangover-sensitive group (*p* < 0.0071, after Bonferroni’s correction for multiple comparisons) were found.

Table A12 and Figure 12 summarize the vomiting ratings of the hangover-resistant group and the hangover-sensitive group. For both the hangover-resistant group and hangover-sensitive group, no significant differences were found between the alcohol and control day. On both test days, the average vomiting rating of the hangover-resistant group remained below 2 (out of 10), and the average vomiting rating for the hangover-sensitive group remained below 1. On both the control day and the alcohol day, no significant differences in vomiting ratings were found between the hangover-resistant group and the hangover-sensitive group.

### 3.18. Shivering

**Figure 13:**
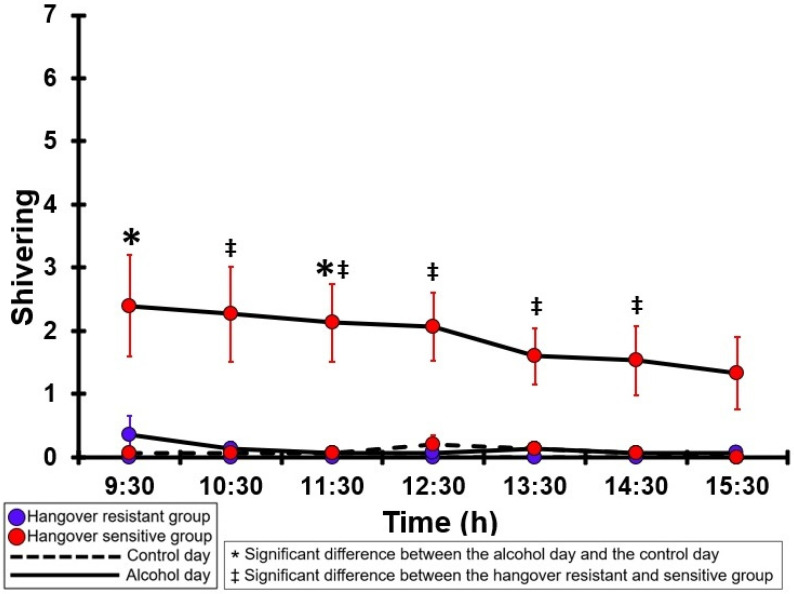
Shivering. Mean and standard error (SE) are shown. Significant differences (at the same timepoint) between the control day and the alcohol day (*p* < 0.0071, after Bonferroni’s correction for multiple comparisons) are indicated by *. Significant differences (at the same timepoint) between the hangover-resistant group and the hangover-sensitive group (*p* < 0.0071, after Bonferroni’s correction for multiple comparisons) are indicated by ‡.

Table A13 and Figure 13 summarize the shivering ratings of the hangover-resistant group and the hangover-sensitive group. For the hangover-resistant group, no significant differences were found between the alcohol and control days. On both test days, the average shivering ratings of the hangover-resistant group remained below 1 (out of 10). For the hangover-sensitive group, the shivering rating at 09:30 on the alcohol day was 2.4 and then gradually decreased during the day. Significantly higher shivering ratings on the alcohol day compared to the control day were found at 09:30 and 11:30. On the control day, no significant differences in shivering ratings were found between the hangover-resistant group and the hangover-sensitive group. On the alcohol day, the shivering ratings of the hangover-sensitive group were significantly higher than those of the hangover-resistant group from 10:30 to 14:30. 

### 3.19. Dizziness

**Figure 14:**
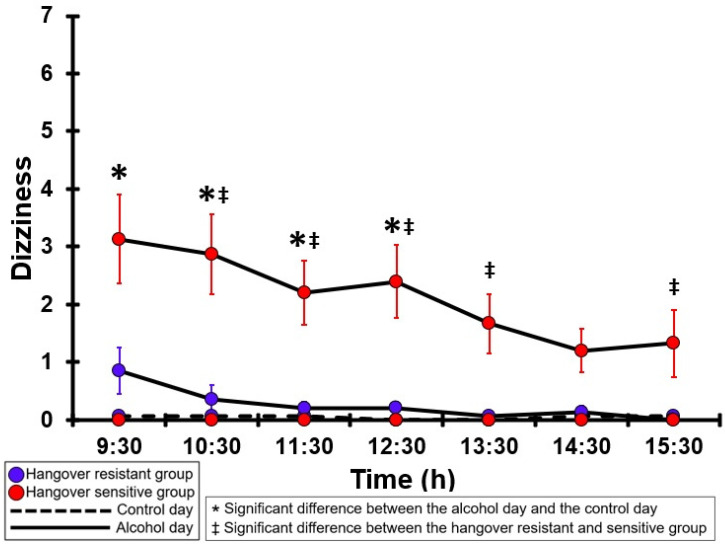
Dizziness. Mean and standard error (SE) are shown. Significant differences (at the same timepoint) between the control day and the alcohol day (*p* < 0.0071, after Bonferroni’s correction for multiple comparisons) are indicated by *. Significant differences (at the same timepoint) between the hangover-resistant group and the hangover-sensitive group (*p* < 0.0071, after Bonferroni’s correction for multiple comparisons) are indicated by ‡.

Table A14 and Figure 14 summarize the dizziness ratings of the hangover-resistant group and the hangover-sensitive group. For the hangover-resistant group, no significant differences were found between the alcohol and control days. On both test days, the average dizziness ratings of the hangover-resistant group remained below 1 (out of 10). For the hangover-sensitive group, the dizziness rating at 09:30 on the alcohol day was 3.1 and then gradually decreased during the day. Significantly higher dizziness ratings on the alcohol day compared to the control day were found from 09:30 to 12:30. On the control day, no significant differences in dizziness ratings were found between the hangover-resistant group and the hangover-sensitive group. On the alcohol day, the dizziness ratings of the hangover-sensitive group were significantly higher than those of the hangover-resistant group at each timepoint except 09:30 and 14:30. 

### 3.20. Sensitivity to Light

**Figure 15:**
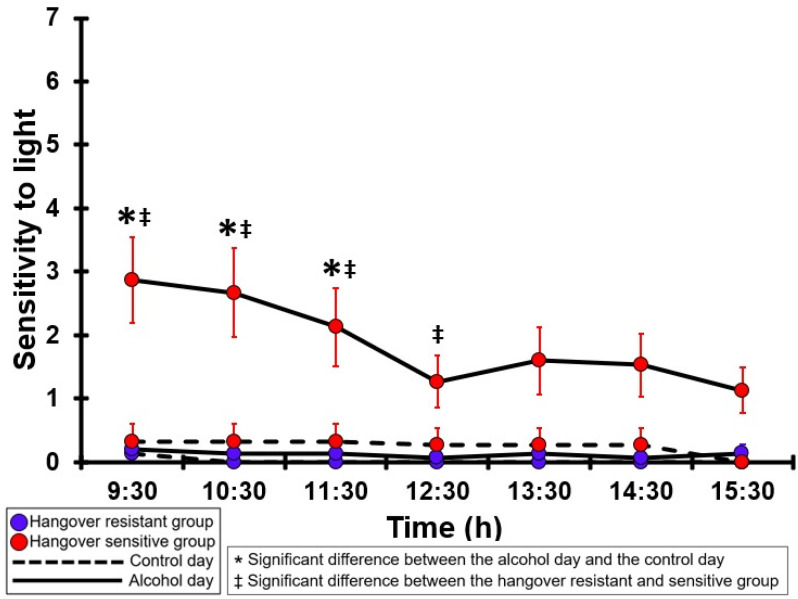
Sensitivity to light. Mean and standard error (SE) are shown. Significant differences (at the same timepoint) between the control day and the alcohol day (*p* < 0.0071, after Bonferroni’s correction for multiple comparisons) are indicated by *. Significant differences (at the same timepoint) between the hangover-resistant group and the hangover-sensitive group (*p* < 0.0071, after Bonferroni’s correction for multiple comparisons) are indicated by ‡.

Table A15 and Figure 15 summarize the sensitivity to light ratings of the hangover-resistant group and the hangover-sensitive group. For the hangover-resistant group, no significant differences were found between the alcohol and control days. On both test days, the average sensitivity to light ratings of the hangover-resistant group remained below 1 (out of 10). For the hangover-sensitive group, the sensitivity to light rating at 09:30 on the alcohol day was 2.9 and then gradually decreased during the day. Significantly higher sensitivity to light ratings on the alcohol day compared to the control day were found from 09:30 to 11:30. On the control day, no significant differences in the sensitivity to light ratings were found between the hangover-resistant group and the hangover-sensitive group. On the alcohol day, the sensitivity to light ratings of the hangover-sensitive group were significantly higher than those of the hangover-resistant group from 09:30 to 12:30. 

### 3.21. Sensitivity to Sound

**Figure 16:**
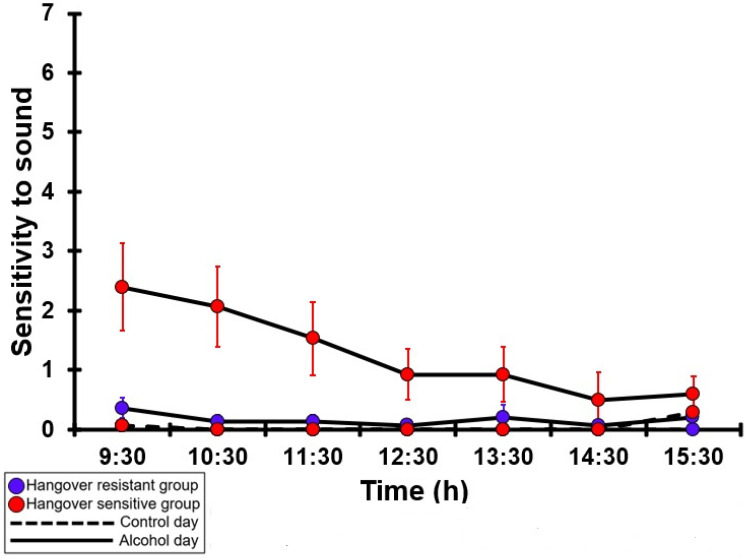
Sensitivity to sound. Mean and standard error (SE) are shown. No significant differences (at the same timepoint) between the control day and the alcohol day (*p* < 0.0071, after Bonferroni’s correction for multiple comparisons) were found. No significant differences (at the same timepoint) between the hangover-resistant group and the hangover-sensitive group (*p* < 0.0071, after Bonferroni’s correction for multiple comparisons) were found.

Table A16 and Figure 16 summarize the sensitivity to sound ratings of the hangover-resistant group and the hangover-sensitive group. For both the hangover-resistant group and the hangover-sensitive group, no significant differences were found between the alcohol and control days. On both test days, the average sensitivity to sound ratings of the hangover-resistant group remained below 1 (out of 10). For the hangover-sensitive group, the sensitivity to sound rating at 09:30 on the alcohol day was 2.4 and then gradually decreased during the day. On both the control day and the alcohol day, no significant differences in sensitivity to sound ratings were found between the hangover-resistant group and the hangover-sensitive group.

### 3.22. Sweating

**Figure 17:**
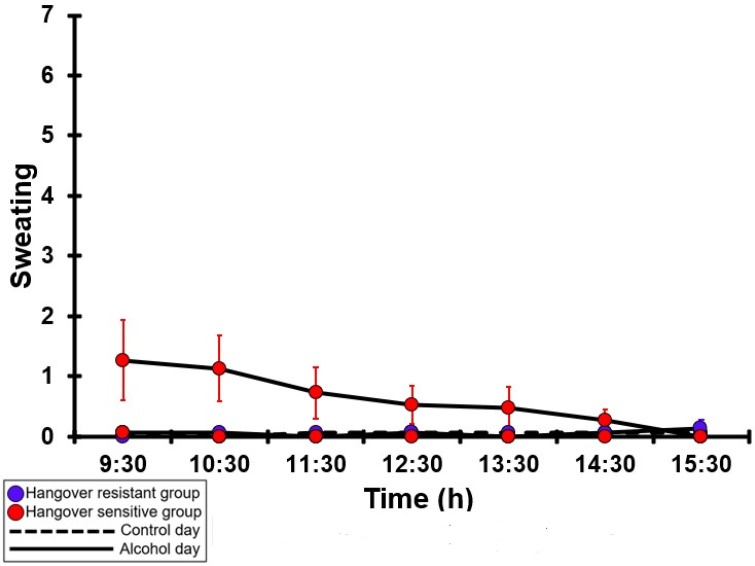
Sweating. Mean and standard error (SE) are shown. No significant differences (at the same timepoint) between the control day and the alcohol day (*p* < 0.0071, after Bonferroni’s correction for multiple comparisons) were found. No significant differences (at the same timepoint) between the hangover-resistant group and the hangover-sensitive group (*p* < 0.0071, after Bonferroni’s correction for multiple comparisons) were found.

Table A17 and Figure 17 summarize the sweating ratings of the hangover-resistant group and the hangover-sensitive group. For both the hangover-resistant group and the hangover-sensitive group, no significant differences were found between the alcohol and control days. On both test days, the average sweating ratings of the hangover-resistant group remained below 1 (out of 10). For the hangover-sensitive group, the sweating rating at 09:30 on the alcohol day was 1.3 and then gradually decreased during the day. On both the control day and the alcohol day, no significant differences in sweating ratings were found between the hangover-resistant group and the hangover-sensitive group. 

### 3.23. Heart Pounding

**Figure 18:**
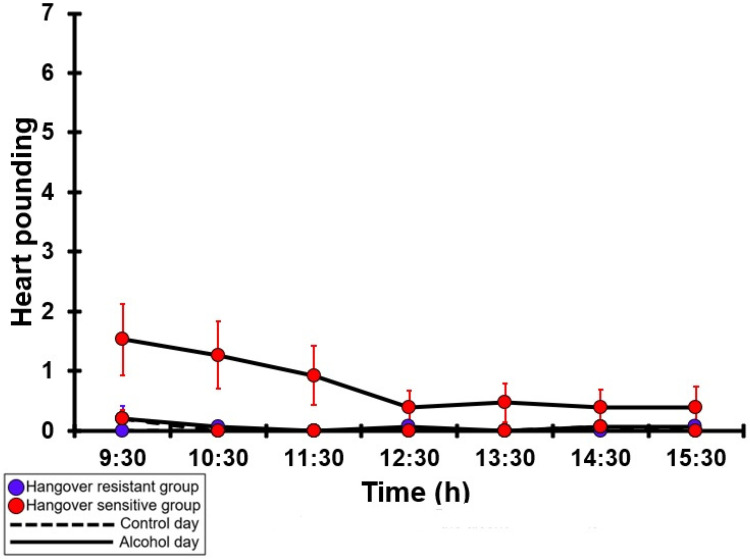
Heart pounding. Mean and standard error (SE) are shown. No significant differences (at the same timepoint) between the control day and the alcohol day (*p* < 0.0071, after Bonferroni’s correction for multiple comparisons) were found. No significant differences (at the same timepoint) between the hangover-resistant group and the hangover-sensitive group (*p* < 0.0071, after Bonferroni’s correction for multiple comparisons) were found.

Table A18 and Figure 18 summarize the heart-pounding ratings of the hangover-resistant group and the hangover-sensitive group. For both the hangover-resistant group and the hangover-sensitive group, no significant differences were found between the alcohol and control day. On both test days, the average heart-pounding ratings of the hangover-resistant group remained below 1 (out of 10). For the hangover-sensitive group, the heart-pounding rating at 09:30 on the alcohol day was 1.5 and then gradually decreased during the day. On both the control day and the alcohol day, no significant differences in heart-pounding ratings were found between the hangover-resistant group and the hangover-sensitive group. 

### 3.24. Heart Racing

**Figure 19:**
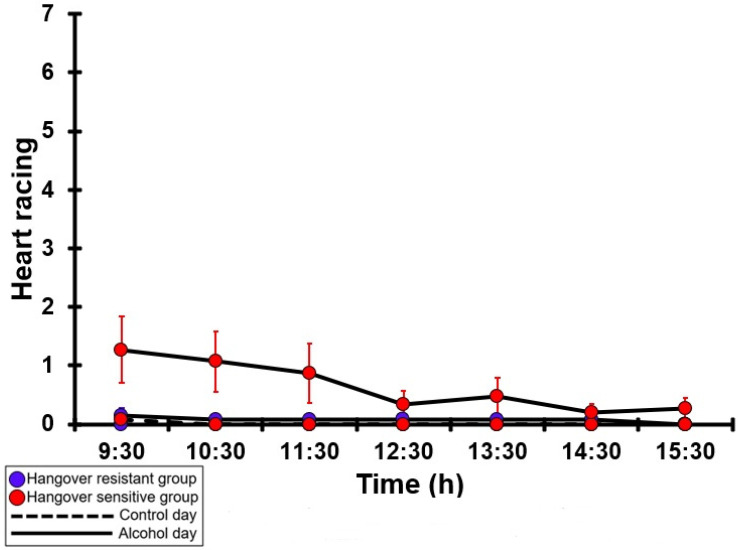
Heart racing. Mean and standard error (SE) are shown. No significant differences (at the same timepoint) between the control day and the alcohol day (*p* < 0.0071, after Bonferroni’s correction for multiple comparisons) were found. No significant differences (at the same timepoint) between the hangover-resistant group and the hangover-sensitive group (*p* < 0.0071, after Bonferroni’s correction for multiple comparisons) were found.

Table A19 and Figure 19 summarize the heart-racing ratings of the hangover-resistant group and the hangover-sensitive group. For both the hangover-resistant group and the hangover-sensitive group, no significant differences were found between the alcohol and control days. On both test days, the average heart-racing ratings of the hangover-resistant group remained below 1 (out of 10). For the hangover-sensitive group, the heart racing rating at 09:30 on the alcohol day was 1.3 and then gradually decreased during the day. On both the control day and the alcohol day, no significant differences in heart-racing ratings were found between the hangover-resistant group and the hangover-sensitive group. 

### 3.25. Thirst

**Figure 20:**
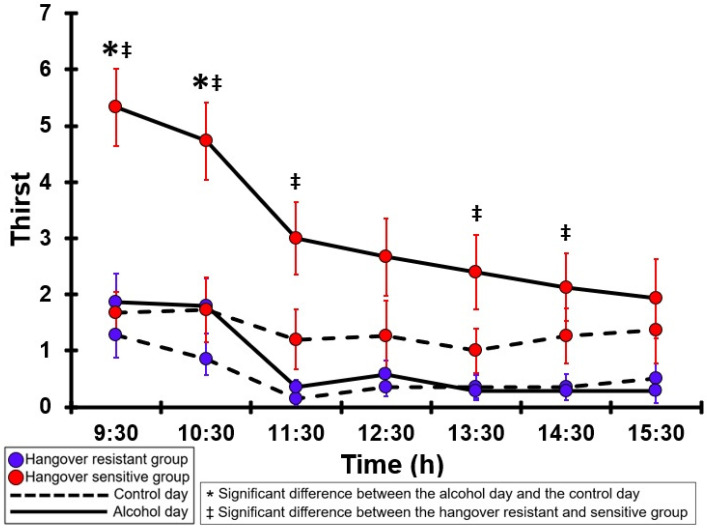
Thirst. Mean and standard error (SE) are shown. Significant differences (at the same timepoint) between the control day and the alcohol day (*p* < 0.0071, after Bonferroni’s correction for multiple comparisons) are indicated by *. Significant differences (at the same timepoint) between the hangover-resistant group and the hangover-sensitive group (*p* < 0.0071, after Bonferroni’s correction for multiple comparisons) are indicated by ‡.

Table A20 and Figure 20 summarize the thirst ratings of the hangover-resistant group and the hangover-sensitive group. For the hangover-resistant group, no significant differences were found between the alcohol and control days. On both test days, the average thirst ratings of the hangover-resistant group remained below 2 (out of 10). For the hangover-sensitive group, the thirst rating at 09:30 on the alcohol day was 5.3 and then gradually decreased during the day. Significantly higher thirst ratings on the alcohol day compared to the control day were found at 09:30 and 10:30. On the control day, no significant differences in thirst ratings were found between the hangover-resistant group and the hangover-sensitive group. On the alcohol day, the thirst ratings of the hangover-sensitive group were significantly higher than those of the hangover-resistant group from 09:30 to 11:30 and at 13:30 and 14:30.

### 3.26. Regret

**Figure 21:**
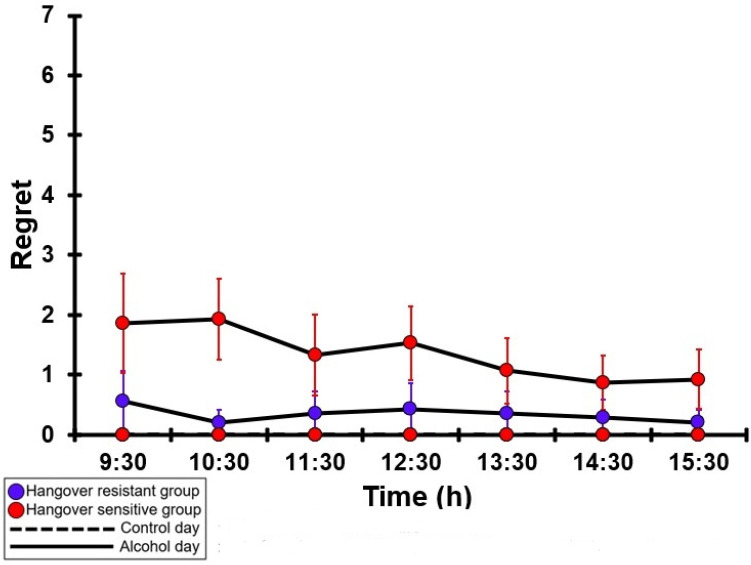
Regret. Mean and standard error (SE) are shown. No significant differences (at the same timepoint) between the control day and the alcohol day (*p* < 0.0071, after Bonferroni’s correction for multiple comparisons) were found. No significant differences (at the same timepoint) between the hangover-resistant group and hangover-sensitive group (*p* < 0.0071, after Bonferroni’s correction for multiple comparisons) were found.

Table A21 and Figure 21 summarize the regret ratings of the hangover-resistant group and the hangover-sensitive group. For both the hangover-resistant group and the hangover-sensitive group, no significant differences were found between the alcohol and control days. On both test days, the average regret ratings of the hangover-resistant group remained below 1 (out of 10). For the hangover-sensitive group, the regret rating at 09:30 on the alcohol day was 1.9 and then gradually decreased during the day. On both the control day and the alcohol day, no significant differences in regret ratings were found between the hangover-resistant group and the hangover-sensitive group. 

### 3.27. Anxiety

**Figure 22:**
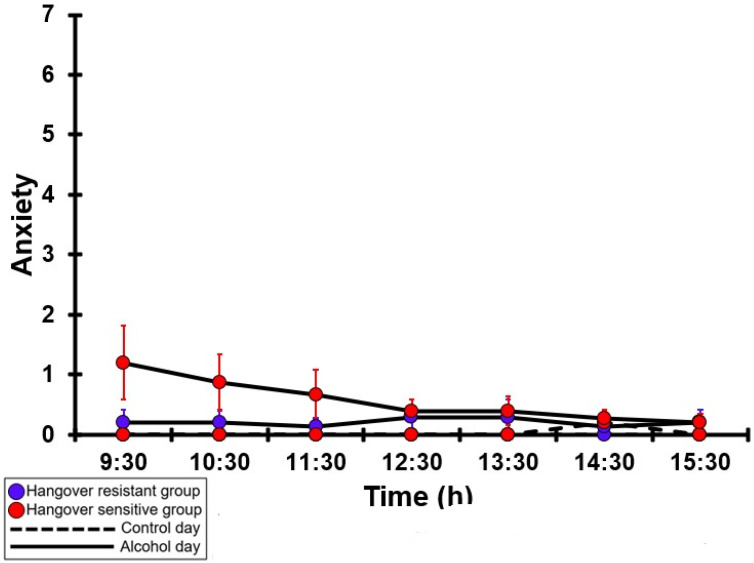
Anxiety. Mean and standard error (SE) are shown. No significant differences (at the same timepoint) between the control day and the alcohol day (*p* < 0.0071, after Bonferroni’s correction for multiple comparisons) were found. No significant differences (at the same timepoint) between the hangover-resistant group and the hangover-sensitive group (*p* < 0.0071, after Bonferroni’s correction for multiple comparisons) were found.

Table A22 and Figure 22 summarize the anxiety ratings of the hangover-resistant group and the hangover-sensitive group. For both the hangover-resistant group and the hangover-sensitive group, no significant differences were found between the alcohol and control days. On both test days, the average anxiety ratings of the hangover-resistant group remained below 1 (out of 10). For the hangover-sensitive group, the anxiety rating at 09:30 on the alcohol day was 1.2 and then gradually decreased during the day. On both the control day and the alcohol day, no significant differences in anxiety ratings were found between the hangover-resistant group and the hangover-sensitive group.

### 3.28. Depression

**Figure 23:**
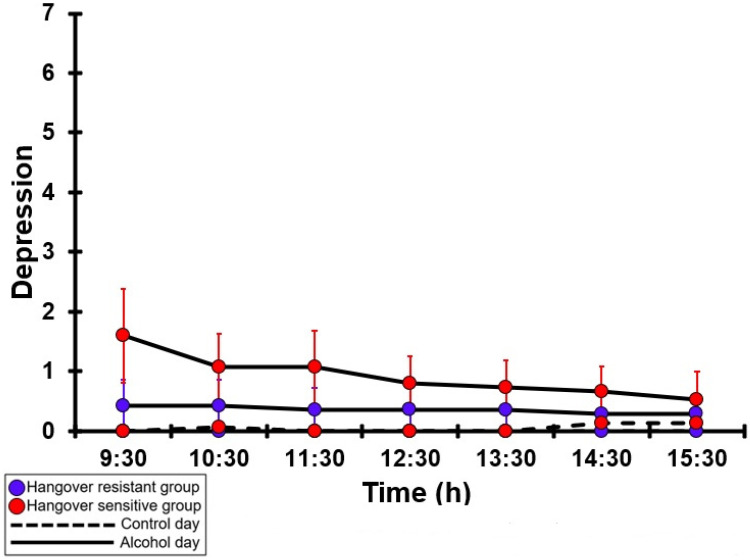
Depression. Mean and standard error (SE) are shown. No significant differences (at the same timepoint) between the control day and the alcohol day (*p* < 0.0071, after Bonferroni’s correction for multiple comparisons) were found. No significant differences (at the same timepoint) between the hangover-resistant group and the hangover-sensitive group (*p* < 0.0071, after Bonferroni’s correction for multiple comparisons) were found.

Table A23 and Figure 23 summarize the depression ratings of the hangover-resistant group and the hangover-sensitive group. For both the hangover-resistant group and the hangover-sensitive group, no significant differences were found between the alcohol and control days. On both test days, the average depression ratings of the hangover-resistant group remained below 1 (out of 10). For the hangover-sensitive group, the depression rating at 09:30 on the alcohol day was 1.2 and then gradually decreased during the day. On both the control day and the alcohol day, no significant differences in depression ratings were found between the hangover-resistant group and the hangover-sensitive group. 

### 3.29. Reduced Appetite

**Figure 24:**
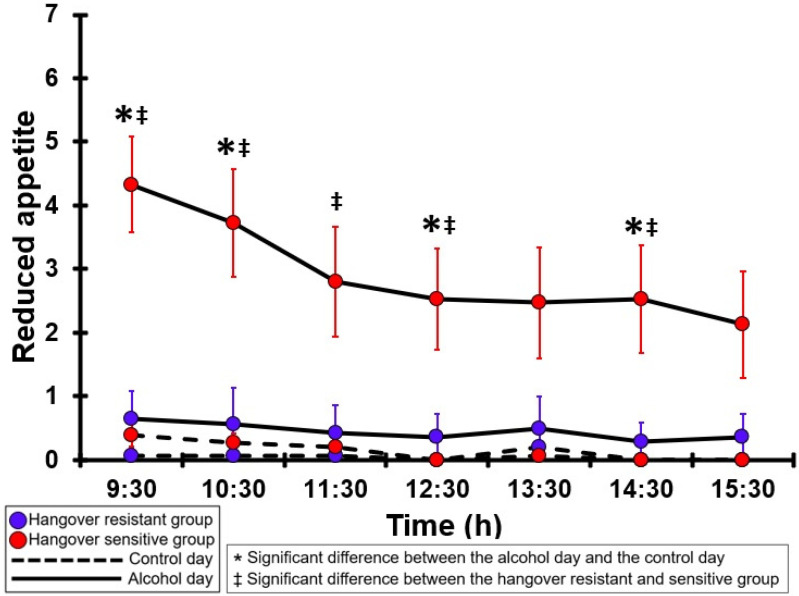
Reduced appetite. Mean and standard error (SE) are shown. Significant differences (at the same timepoint) between the control day and the alcohol day (*p* < 0.0071, after Bonferroni’s correction for multiple comparisons) are indicated by *. Significant differences (at the same timepoint) between the hangover-resistant group and the hangover-sensitive group (*p* < 0.0071, after Bonferroni’s correction for multiple comparisons) are indicated by ‡.

Table A24 and Figure 24 summarize the reduced appetite ratings of the hangover-resistant group and the hangover-sensitive group. For the hangover-resistant group, no significant differences were found between the alcohol and control days. On both test days, the average reduced appetite ratings of the hangover-resistant group remained below 1 (out of 10). For the hangover-sensitive group, the reduced appetite rating at 09:30 on the alcohol day was 4.3 and then gradually decreased during the day. Significantly higher reduced appetite ratings on the alcohol day compared to the control day were found at 09:30, 10:30, 12:30, and 14:30. On the control day, no significant differences in reduced appetite ratings were found between the hangover-resistant group and the hangover-sensitive group. On the alcohol day, reduced appetite ratings of the hangover-sensitive group were significantly higher than those of the hangover-resistant group from 09:30 to 12:30 and at 14:30. 

### 3.30. Karoliska Sleepiness Scale (KSS)

**Figure 25:**
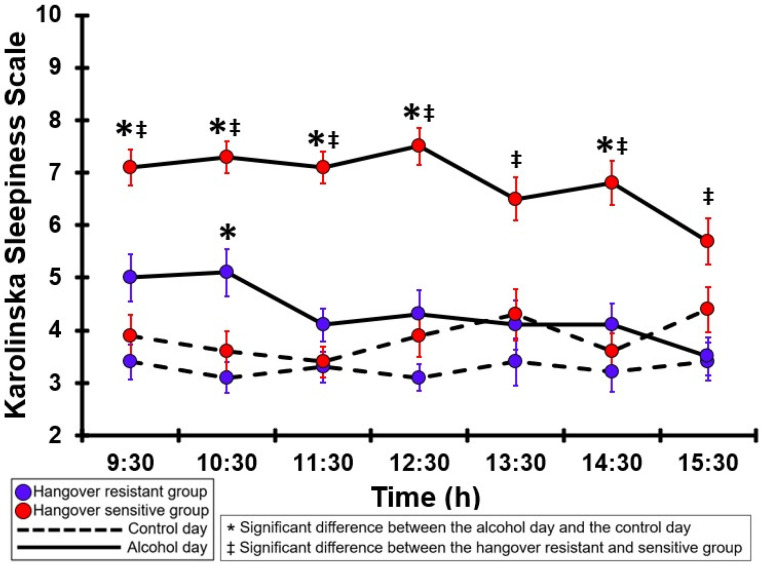
Karolinska Sleepiness Scale. Mean and standard error (SE) are shown. Significant differences (at the same timepoint) between the control day and the alcohol day (*p* < 0.0071, after Bonferroni’s correction for multiple comparisons) are indicated by *. Significant differences (at the same timepoint) between the hangover-resistant group and the hangover-sensitive group (*p* < 0.0071, after Bonferroni’s correction for multiple comparisons) are indicated by ‡.

Table A25 and Figure 25 summarize the KSS sleepiness ratings of the hangover-resistant group and the hangover-sensitive group. For the hangover-resistant group, KSS sleepiness ratings were higher on the alcohol day than on the control day; however, the difference was statistically significant only at 10:30. On both test days, KSS sleepiness ratings of the hangover-resistant group remained below 6 (out of 10). For the hangover-sensitive group, the KSS sleepiness rating at 09:30 on the alcohol day was 7.1, remained high until 12:30, and then gradually decreased. Significantly higher KSS sleepiness ratings on the alcohol day compared to the control day were found at all timepoints except 13:30 and 15:30. On the control day, no significant differences in KSS sleepiness ratings were found between the hangover-resistant group and the hangover-sensitive group. On the alcohol day, at all timepoints the KSS sleepiness ratings of the hangover-sensitive group were significantly higher than those of the hangover-resistant group. 

### 3.31. POMS-SF—Depression

**Figure 26:**
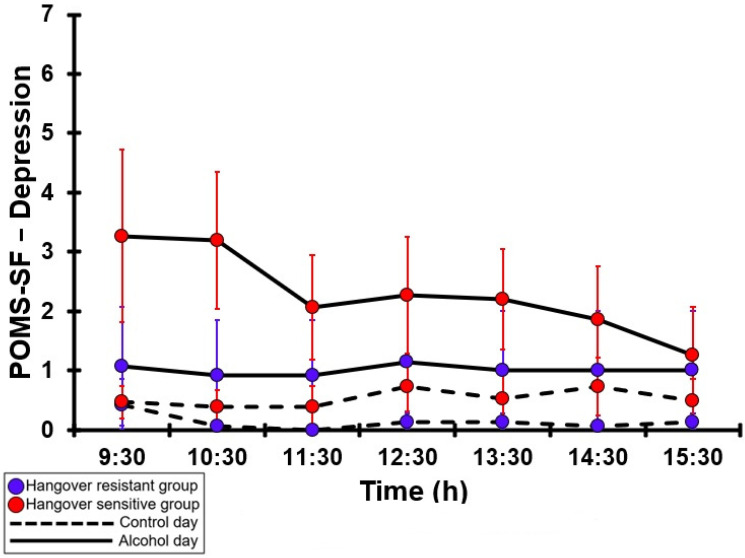
POMS-SF—Depression. No significant differences (at the same timepoint) between the control day and the alcohol day (*p* < 0.0071, after Bonferroni’s correction for multiple comparisons) were found. No significant differences (at the same timepoint) between the hangover-resistant group and the hangover-sensitive group (*p* < 0.0071, after Bonferroni’s correction for multiple comparisons) were found.

Table A26 and Figure 26 summarize the POMS-SF—Depression ratings of the hangover-resistant group and the hangover-sensitive group. For both groups, no significant differences were found between the alcohol day and the control day. On both test days, the POMS-SF—Depression ratings of both groups remained below 4 (out of 32). On both the control day and the alcohol day, no significant differences were found between the hangover-resistant group and the hangover-sensitive group. 

### 3.32. POMS-SF—Anger

**Figure 27:**
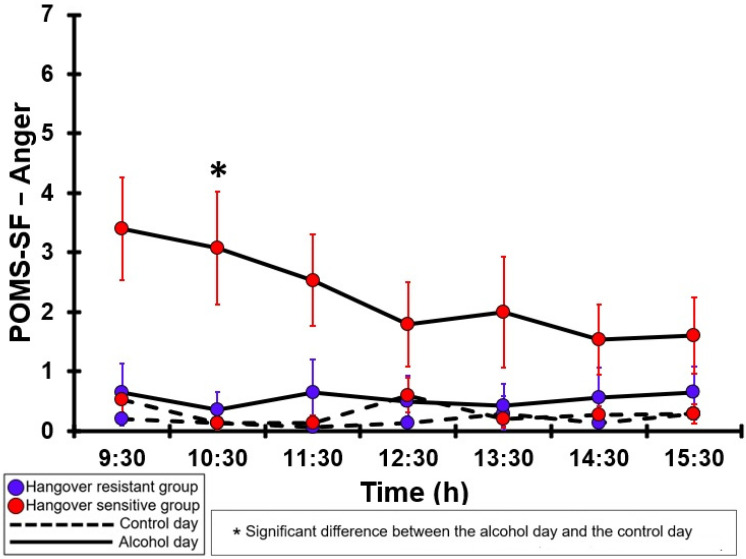
POMS-SF—Anger. Significant differences (at the same timepoint) between the control day and the alcohol day (*p* < 0.0071, after Bonferroni’s correction for multiple comparisons) are indicated by *. No significant differences (at the same timepoint) between the hangover-resistant group and the hangover-sensitive group (*p* < 0.0071, after Bonferroni’s correction for multiple comparisons) were found.

Table A27 and Figure 27 summarize the POMS-SF—Anger ratings of the hangover-resistant group and the hangover-sensitive group. On both test days, the POMS-SF—Anger ratings of both groups remained below 1 (out of 28). For the hangover-resistant group, no significant differences were found between the alcohol day and the control day. For the hangover-sensitive group, the POMS-SF—Anger rating at 09:30 on the alcohol day was 3.4, and then gradually decreased during the day. However, a significant increase in POMS-SF—Anger ratings was found only at 10:30. On both the control day and the alcohol day, no significant differences were found between the hangover-resistant group and the hangover-sensitive group. 

### 3.33. POMS-SF—Tension 

**Figure 28:**
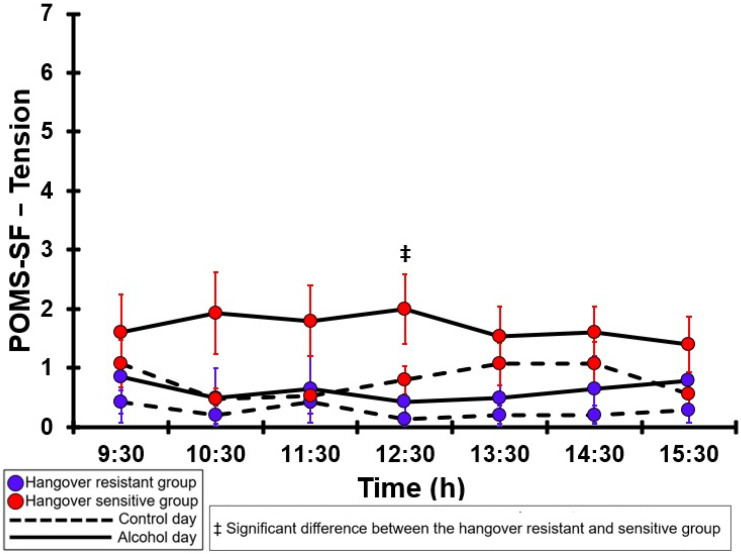
POMS-SF—Tension. No significant differences (at the same timepoint) between the control day and the alcohol day (*p* < 0.0071, after Bonferroni’s correction for multiple comparisons) were found. Significant differences (at the same timepoint) between the hangover-resistant group and the hangover-sensitive group (*p* < 0.0071, after Bonferroni’s correction for multiple comparisons) are indicated by ‡.

Table A28 and Figure 28 summarize the POMS-SF—Tension ratings of the hangover-resistant group and the hangover-sensitive group. On both test days, the POMS-SF—Tension ratings of both groups were ≤2 (out of 24). For both the hangover-resistant group and the hangover-sensitive group, no significant differences were found between the alcohol day and the control day. On the control day, no significant differences were found between the groups. For the alcohol day, the POMS-SF—Tension rating of the hangover-sensitive group was significantly higher than that of the hangover-resistant group at 12:30, but no significant differences between the groups were found at other timepoints. 

### 3.34. POMS-SF—Fatigue

**Figure 29:**
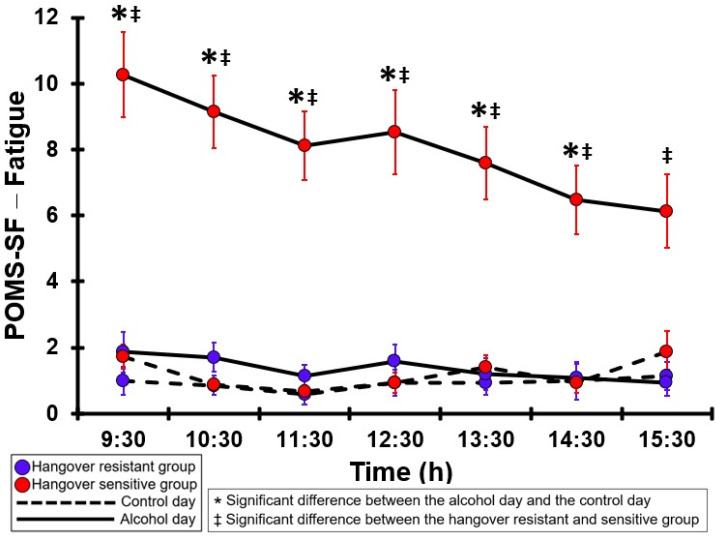
POMS-SF—Fatigue. Significant differences (at the same timepoint) between the control day and the alcohol day (*p* < 0.0071, after Bonferroni’s correction for multiple comparisons) are indicated by *. Significant differences (at the same timepoint) between the hangover-resistant group and the hangover-sensitive group (*p* < 0.0071, after Bonferroni’s correction for multiple comparisons) are indicated by ‡.

Table A29 and Figure 29 summarize the POMS-SF—Fatigue ratings of the hangover-resistant group and the hangover-sensitive group. On both test days, the POMS-SF—Fatigue ratings of the hangover-resistant group were below 2 (out of 24). For the hangover-sensitive group, the POMS-SF—Fatigue rating at 09:30 on the alcohol day was 10.3 and then gradually decreased during the day. Significantly higher POMS-SF—Fatigue ratings on the alcohol day compared to the control day were found at all timepoints except 15:30. On the control day, no significant differences in POMS-SF—Fatigue ratings were found between the hangover-resistant group and the hangover-sensitive group. On the alcohol day, at all timepoints POMS-SF—Fatigue ratings of the hangover-sensitive group were significantly higher than those of the hangover-resistant group. 

### 3.35. POMS-SF—Vigor

**Figure 30:**
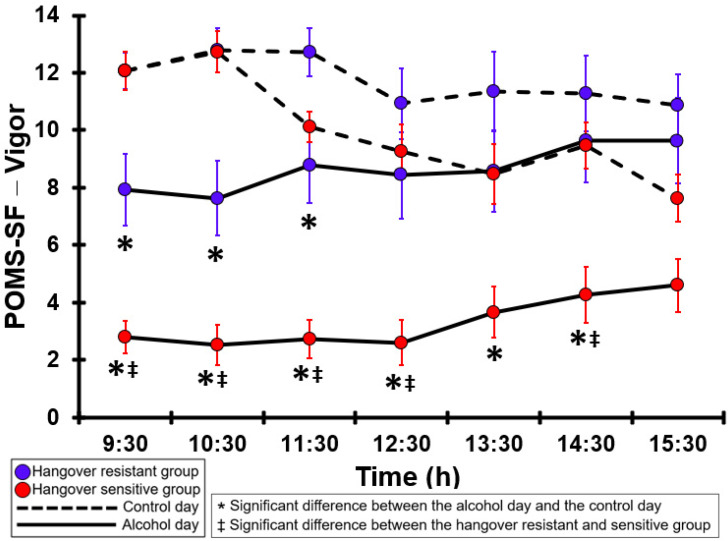
POMS-SF—Vigor. Significant differences (at the same timepoint) between the control day and the alcohol day (*p* < 0.0071, after Bonferroni’s correction for multiple comparisons) are indicated by *. Significant differences (at the same timepoint) between the hangover-resistant group and the hangover-sensitive group (*p* < 0.0071, after Bonferroni’s correction for multiple comparisons) are indicated by ‡.

Table A30 and Figure 30 summarize the POMS-SF—Vigor ratings of the hangover-resistant group and the hangover-sensitive group. For the hangover-resistant group, on both test days, the POMS-SF—Vigor ratings were highest in the morning and then gradually decreased during the day. From 09:30 to 11:30 the POMS-SF—Vigor ratings on the alcohol day were significantly lower compared to the control day. For the hangover-sensitive group, the POMS-SF—Vigor ratings of the control day did not significantly differ from those of the hangover-resistant group. However, the POMS-SF—Vigor rating at 09:30 on the alcohol day was 2.8 and then gradually increased to 4.6 at 15:30. Significantly lower POMS-SF—Vigor ratings on the alcohol day compared to the control day were found at all timepoints except 15:30. On the alcohol day, at all timepoints except 13:30 and 15:30 the POMS-SF—Vigor ratings of the hangover-sensitive group were significantly lower than those of the hangover-resistant group. 

### 3.36. Predictors of Overall Hangover Severity

**Figure A1:**
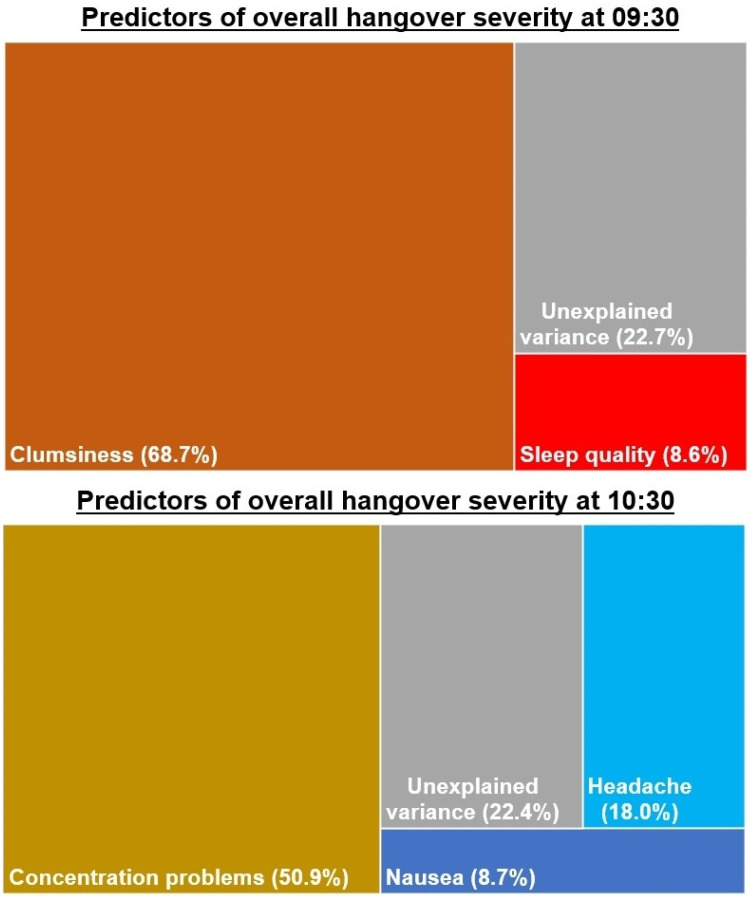
Predictors of overall hangover severity in the morning.

**Figure A2:**
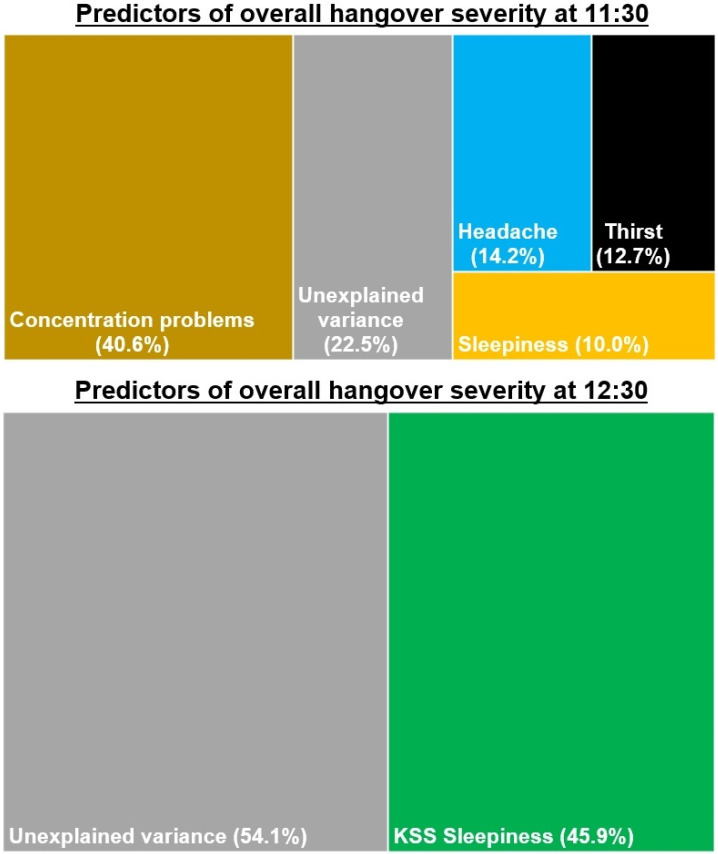
Predictors of overall hangover severity at different timepoints at midday.

**Figure A3:**
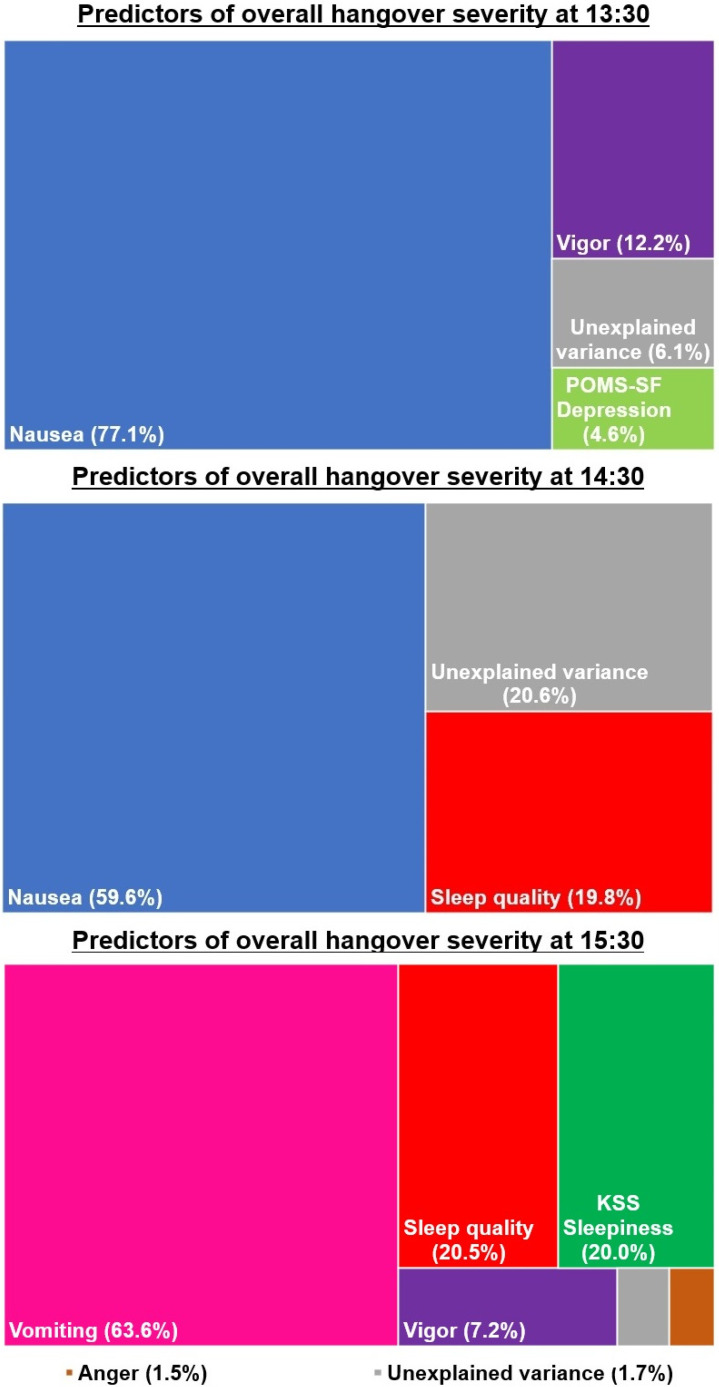
Predictors of overall hangover severity at different timepoints in the afternoon.

It is evident from the previous sections that both the overall hangover severity and the severity of individual items reported by hangover-sensitive drinkers vary during the day. Overall, the intensity of symptoms is greatest in the morning assessments and then gradually declines during the day. There is, however, a difference in the rate of decline between symptoms, and whereas the severity of some symptoms is still significantly greater at 15:30 on the alcohol day compared to the control day, this is not the case for other symptoms. With the latter in mind, a series of stepwise linear regression analyses were conducted to identify predictors of overall hangover severity for each timepoint. Only participants of the hangover-sensitive group were included. Included independent variables were all 23 individual symptoms, KSS sleepiness, the POMS-SF mood subscales, sleep quality (assessed with the Groningen Sleep Quality Scale), the amount of alcohol consumed, and estimated BAC. The results of the analyses for the morning, midday, and afternoon assessments are summarized in Figure A1, Figure A2 and Figure A3, respectively.

Comparing the analyses of the different timepoints reveals that in the morning and midday, the predictors that explain the most variance in overall hangover severity are clumsiness, concentration problems, and headache (See Figure A1 and Figure A2). In contrast, in the afternoon the most contributing predictors include nausea and vomiting, sleepiness (assessed with the KSS), and previous night sleep quality (See Figure A3). The outcomes confirm that previous night sleep quality, daytime sleepiness, headache, and nausea are core determinants of overall hangover severity.

Interestingly, mood factors, either assessed as individual items or via the POMS-SF, have no or only a very small contribution to the models that predict overall hangover severity. This finding is in line with the fact that mood changes during the hangover state are usually only modest and reported by a minority of drinkers [4]. Also of interest are the findings regarding thirst. Although thirst is one of the most frequently reported symptoms with a high severity score, only at one timepoint (11:30) thirst appeared to be a minor determinant of overall hangover severity (contributing 12.7% to the model).

## 4. Discussion

In this semi-naturalistic study, a comprehensive and detailed comparison was made between the next-day effects of an evening of alcohol consumption between hangover-sensitive drinkers and hangover-resistant drinkers. As a start, it is important to note that the two groups did not significantly differ from each other on demographic characteristics and baseline sleep. On the alcohol test day, both groups consumed a considerable amount of alcohol that should be sufficient to elicit a hangover in sensitive drinkers. Of importance for a fair comparison between the hangover-sensitive group and the hangover-resistant group, no significant differences were found in the amount of alcohol consumed on the alcohol day, the estimated BAC, and the start and stop time of alcohol consumption. The groups did also not differ in reported activities on the test days. Both groups slept significantly shorter on the alcohol day compared to the control day. Although no significant differences were found on any of the sleep outcomes (e.g., time to bed, total sleep time) between the two groups on either the alcohol or the control day, the hangover-sensitive group did report a significantly poorer sleep quality on the alcohol day compared to the hangover-resistant group. The morning assessments also reported no clinically relevant differences between the alcohol and control day or between the groups for anxiety (STAI-Y), depression (BDI-II), and risk-taking behavior (RT-18). Taken together, a fair comparison between the next-day effects of an evening of alcohol consumption between the hangover-sensitive group and the hangover-resistant group is warranted. 

**Figure 31:**
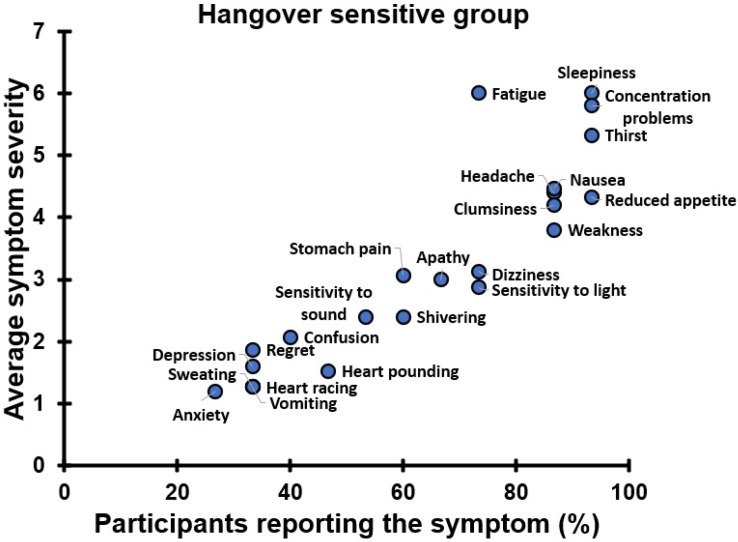
Overview of reported next-day symptoms at 09:30 by the hangover-sensitive group on the alcohol day.

The outcomes of the next-day symptom assessments revealed significant differences between the two groups. In the hangover-sensitive group, a variety of symptoms were reported the day following alcohol consumption. Their presence and severity are summarized in Figure 31.

The most frequently reported symptoms by hangover-sensitive drinkers at 09:30 are sleepiness, concentration problems, and thirst. These symptoms had also the highest severity scores. Other symptoms with high presence and severity were fatigue, clumsiness, weakness, nausea, and headache. The least frequently reported symptom, with the lowest severity score, was anxiety. The severity of symptoms declined throughout the day. However, for most symptoms throughout the day, significantly higher severity scores were reported on the alcohol day compared to the control day. The KSS assessment revealed a significantly increased sleepiness throughout the alcohol day. In line, the POMS-SF mood assessments revealed significantly increased fatigue scores and significantly reduced vigor. No significant effects were found for depression and tension, whereas a significant increase in anger on the alcohol day was found only at 10:30.

No significant differences between the alcohol and control day were found for confusion, stomach pain, vomiting, sensitivity to sound, sweating, heart pounding, heart racing, regret, anxiety, and depression. On first thought, it could be questioned if these symptoms should be considered hangover-related. Alternatively, one could argue that these symptoms are indeed less frequently reported, but that severity scores of these symptoms by those who do report them are comparable to those of other symptoms. 

**Figure 32:**
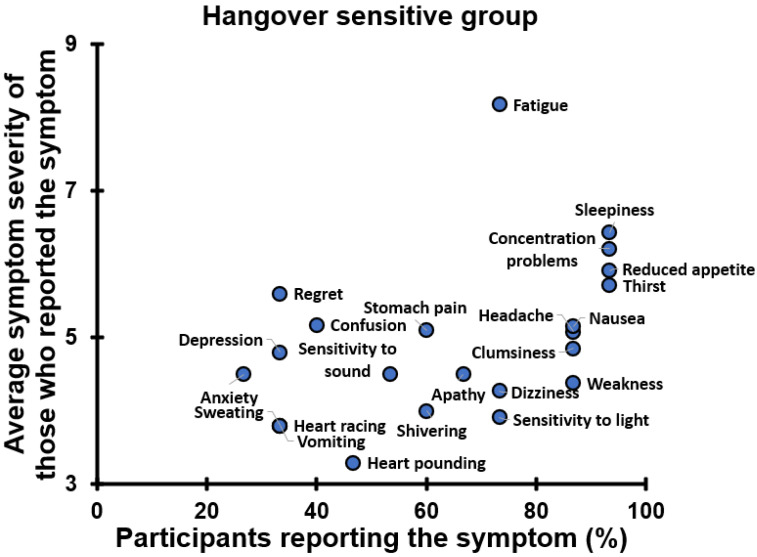
Average severity of next-day symptoms at 09:30 by those who reported the corresponding symptom.

Figure 32 shows the average severity scores of only those participants of the hangover-sensitive group that reported the corresponding symptom. It is evident that the severity scores of most symptoms are in the range of 3 to 6, corresponding to moderate intensity. Given this, one should conclude that these symptoms are also relevant, though less frequently reported. Whereas research usually reports results at the group level (e.g., averages), the data presented in Figure 32 advocate that it is also important to look at individual study participants. Whereas at the group level, an average symptom may be low, individual drinkers may experience such symptoms with high intensity.

**Figure 33:**
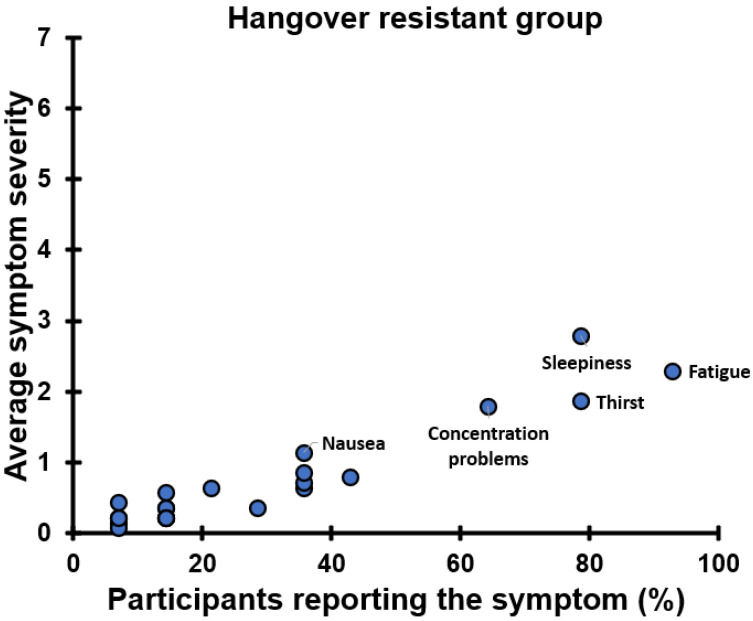
Overview of reported next-day symptoms at 09:30 by the hangover-resistant group on the alcohol day. To enhance readability, symptom labels with severity scores below 1 are omitted from the figure.

The presence and severity of most next-day symptoms of the hangover-resistant group are summarized in Figure 33. In contrast, none of the next-day symptoms at 09:30 reported by the hangover-resistant group differed significantly between the alcohol and control days. At other timepoints, only fatigue at 10:30 was significantly higher on the alcohol day (1.64) than on the control day (0.57). Most symptom scores were below 1 on the 0 to 10 severity scale. The presence and severity of most next-day symptoms were significantly lower compared to those reported by the hangover-sensitive group. 

On the KSS, sleepiness scores on the alcohol day were significantly increased at 10:30. At other timepoints, the increase did not reach statistical significance. Regarding the POMS-SF mood assessments, no significant differences between the alcohol and control days were found for depression, anger, fatigue, and tension. However, significant reductions in vigor were reported on the alcohol day at 09:30, 10:30, and 11:30.

Taken together, the next-day adverse effects reported by hangover-resistant drinkers, if any, are limited to mild sleepiness-related effects and reduced vigor in the morning. These effects can be due to alcohol, but it is more likely that these are related to the sleep loss associated with the alcohol day. 

Our findings are in line with the study conducted by Hogewoning et al. [16]. The current study extends these findings by conducting assessments at multiple timepoints throughout the day. The outcomes of these assessments reveal that symptom severity is most severe in the morning hours and then gradually decreases. However, in the afternoon at 15:30, hangover-sensitive drinkers still report a great number of symptoms that are significantly more severe on the alcohol day compared to the control day. Moreover, at that time, overall hangover severity is still evident (a score of 3.2 out of 10). These findings demonstrate that hangovers are not limited to the morning hours. Although the intensity of experienced symptoms and overall hangover severity declines during the day, they persist throughout the day.

The observation that symptoms such as sleepiness and concentration problems persist up to 15 h after alcohol consumption may have significant implications for daytime functioning. For example, previous studies found that driving ability is significantly impaired in the hangover state [34,35]. These studies assessed simulated driving performance in the morning. The fact that symptoms persist throughout the day may imply that driving will also be impaired in the afternoon. Up to now, only a few studies investigated alcohol hangover effects at different timepoints during the day, with mixed results on the impact of the hangover state on mood, cognition, and performance of psychometric tests [36,37,38]. Future studies should therefore also assess the impact of alcohol hangovers on daytime performance (e.g., driving, work) at different timepoints during the day. 

An explanation for the differences between hangover-sensitive drinkers and hangover-resistant drinkers may be related to the fact that sleep quality on the alcohol day was reported as significantly poorer by the hangover-sensitive group than the hangover-resistant group. Previous research has linked poor sleep quality to hangover severity and next-day sleepiness-related symptoms [39,40,41,42,43,44,45]. However, poor sleep quality and sleep loss without alcohol consumption may also result in next-day sleepiness, fatigue, and concentration problems [46], and have also been associated with other frequently reported hangover symptoms such as headache [47,48] and nausea [49]. One could also argue that participants perceived their sleep quality as worse *because* they experienced daytime sleepiness-related symptoms at the time they completed the sleep scale in the morning. The latter may be a possible explanation, given the fact that no other significant differences were found between the groups on any of the other sleep outcomes, including total sleep time, time to bed, wake-up time, and the number of nightly awakenings. 

Differences between hangover-sensitive drinkers and hangover-resistant drinkers in outcomes of biomarker assessment that were conducted among participants of the study by Hogewoning et al. [16] may provide an alternative explanation. In this study, saliva and urine were collected in the morning on the alcohol day and the control day. Potential biomarkers were assessed and compared between the hangover-sensitive group and the hangover-resistant group. For both test days, no significant differences between the groups were found for urine concentrations of methanol [50], urine concentrations of ethanol metabolites ethyl glucuronide (EtG) and ethyl sulfate (EtS) [51], and 5-hydroxytryptophol (5-HTOL), 5-hydroxyindoleacetic acid (5-HIAA) and the 5-HTOL/5-HIAA ratio [52]. In both groups, the urine concentrations of these biomarkers were increased the morning after alcohol consumption, but their concentration did not correlate with hangover severity. In contrast, although both groups reported having consumed a comparable amount of alcohol, urine ethanol concentrations in the hangover-resistant group were significantly lower compared to those of the hangover-sensitive group [53]. This finding suggests that hangover-resistant drinkers may have an accelerated alcohol metabolism, which may explain why they experience no hangover symptoms [9]. Another study in a small sample of hangover-sensitive drinkers confirmed that their alcohol breakdown rate was significantly and negatively correlated with hangover severity [10]. Another study [10] found that more oxidative stress, measured as the presence of malondialdehyde in blood, in the first hours after alcohol consumption was associated with reporting *less* severe hangovers, whereas the presence of more malondialdehyde at a later timepoint was associated with having *more* severe hangovers. Thus, a quicker elimination of ethanol was associated with reporting less severe hangovers. However, a direct comparison between hangover-sensitive drinkers and hangover-resistant drinkers revealed no significant differences in alcohol breakdown rate after an alcohol challenge [54]. The amount of alcohol administered in this study was, however, low (resulting in a peak BAC of 0.05%). It might be that differences in alcohol metabolism between hangover-sensitive drinkers and hangover-resistant drinkers manifest only at higher BACs. In addition to alcohol metabolism, other analyses focused on the inflammatory response caused by alcohol. Saliva samples from the participants of the study of Hogewoning et al. [16] were analyzed to assess the concentration of a variety of cytokines [55]. Compared to the control day, on the alcohol day significant increases in saliva interleukin (IL)-6 and IL-10 concentrations were found in both groups. However, no significant differences were found between the groups. Moreover, changes in cytokine concentration (alcohol day–control day) did not significantly correlate with hangover severity [55]. These observations were unexpected, as previous survey research suggested that the perceived immune fitness of hangover-resistant drinkers was significantly better that that reported by hangover-sensitive drinkers [56]. Moreover, another study that conducted cytokine assessments in the blood instead of saliva found significant correlations between changes (alcohol day–control day) in IL-12 and interferon (IFN)-γ with overall hangover severity [57]. The course of biological parameters, including biomarkers of systemic inflammation, hormones, and physiological effects should be further evaluated in future studies.

Taken together, there is presently limited research with inconsistent findings on the role of alcohol metabolism and the inflammatory response to alcohol consumption in the pathology of alcohol hangovers. More research is needed to elucidate the pathology of the hangover and to investigate and explain why some drinkers are hangover resistant while others are hangover sensitive. 

## Figures and Tables

**Table 1:** Demographics and baseline sleep assessments.

	Overall	Hangover-Resistant Group	Hangover-Sensitive Group	*p*-Value
Number of subjects	29	14	15	-
Male/Female ratio	15/14	8/6	7/8	0.715
Age (years)	21.1 (2.0)	21.1 (2.2)	21.1 (1.9)	0.914
Weight (kg)	72.9 (10.6)	74.4 (12.6)	71.5 (8.6)	0.477
Height (m)	1.78 (0.1)	1.80 (0.1)	1.76 (0.1)	0.146
BMI (kg/m^2^)	22.9 (2.1)	22.8 (2.2)	23.1 (2.1)	1.000
Sleep-50				
Apnea	10.9 (2.4)	10.4 (1.8)	11.5 (2.8)	0.331
Insomnia	12.0 (3.7)	12.1 (3.9)	11.9 (3.7)	0.847
Narcolepsy	6.4 (1.3)	6.0 (1.2)	6.7 (1.3)	0.123
RLS/PLMD	4.9 (1.0)	5.1 (1.1)	4.7 (0.9)	0.425
CRSD	5.1 (1.5)	4.9 (1.6)	5.3 (1.3)	0.354
Sleepwalking	3.1 (0.3)	3.1 (0.3)	3.1 (0.3)	0.983
Nightmares	4.0 (5.0)	2.4 (3.7)	5.5 (5.7)	0.217
Impact on functioning	10.8 (2.6)	10.3 (2.6)	11.3 (2.6)	0.270
Sleep quality	7.3 (0.9)	7.2 (1.1)	7.5 (0.7)	0.591
Total sleep time (h)	8.1 (0.7)	8.1 (0.9)	8.0 (0.5)	0.683

Mean and standard deviation (SD) are shown. No significant differences (*p* < 0.05) were found between the hangover-resistant group and the hangover-sensitive group. Abbreviations: BMI = body mass index, CRSD = circadian rhythm sleep disorder, RLS/PLMD = restless legs/periodic limb movement disorder.

**Table 2:** Activity on the test days.

	Hangover-Resistant Group		Hangover-Sensitive Group	
Variable	Control Day	Alcohol Day	Control Day	Alcohol Day
At home (non-active)	13	8	11	6
In pub (non-active)	0	2	1	6
Party (dancing)/sports (active)	1	4	3	3

Number of participants engaged in the activity are listed. No significant differences between the hangover-resistant group and the hangover-sensitive group (*p* < 0.05) were found.

**Table 3:** Estimated alcohol consumption.

Variable	Hangover Resistant	Hangover Sensitive	*p*-Value
Number of alcoholic drinks	13.5 (7.9)	12.4 (4.4)	0.847
Start time drinking (h:m)	19:39 (125.3)	19:56 (75.4)	0.949
Stop time drinking (h:m)	00:36 (84.6)	01:02 (96.1)	0.331
Estimated BAC (%)	0.21 (0.1)	0.20 (0.1)	0.533

Mean and standard deviation (SD) are shown. No significant differences between the hangover-resistant group and the hangover-sensitive group (*p* < 0.05) were found. Abbreviations: BAC = blood alcohol concentration, h:m = hour:minutes.

**Table 4:** Sleep on test days.

Group	Hangover-Resistant Group			Hangover-Sensitive Group			R vs. S Group, *p*-Value	
Variable	Control Day	Alcohol Day	*p*-Value	Control Day	Alcohol Day	*p*-Value	Control Day	Alcohol Day
SQ	1.1 (1.4)	2.9 (2.4)	0.132	1.7 (2.2)	5.1 (1.8)	0.008 *	0.813	0.026 ^‡^
NA	0.4 (0.6)	0.5 (0.8)	0.414	0.5 (0.9)	0.6 (0.9)	0.739	1.000	0.847
TST	446.3 (51.0)	371.2 (62.4)	<0.001 *	413.5 (47.7)	339.1 (76.8)	0.008 *	0.097	0.205
TTB	23:33 (49.2)	01:10 (66.4)	<0.001 *	23:35 (60.5)	01:45 (85.4)	<0.001 *	0.949	0.146
TLO	23:47 (46.1)	01:10 (66.2)	0.002 *	23:54 (53.3)	01:53 (83.1)	<0.001 *	0.715	0.112
TFA	00:00 (46.2)	01:19 (67.1)	0.002 *	00:12 (53.2)	01:58 (85.1)	0.003 *	0.477	0.134
SOL	12.8 (10.2)	8.9 (10.5)	0.181	17.9 (14.4)	4.7 (8.1)	0.010 *	0.252	0.201
WUT	07:28 (20.6)	07:34 (29.2)	0.386	07:19 (37.1)	07:37 (25.2)	0.050	0.377	0.949
TOB	07:36 (22.5)	07:43 (29.3)	0.207	07:32 (36.6)	07:50 (22.3)	0.023 *	0.505	0.813

Mean and standard deviation (SD) are shown. Significant differences between the control day and the alcohol day (*p* < 0.05) are indicated by *. Significant differences between the hangover-resistant group and the hangover-sensitive group (*p* < 0.05) are indicated by ^‡^. Abbreviations: R = hangover-resistant group, S = hangover-sensitive group, SQ = sleep quality (assessed with the Groningen Sleep Quality Scale), NA = number of nightly awakenings, TST = total sleep time (min), TTB = time to bed (hh:mm), TLO = time lights out (hh:mm), TFA = time falling asleep (hh:mm), SOL = sleep onset latency (min), WUT = wake up time (hh:mm), TOB = time out of bed (hh:mm).

**Table 5:** Mood assessments in the morning after arrival.

Group	Hangover-Resistant Group			Hangover-Sensitive Group			R vs. S Group, *p*-Value	
Variable	Control Day	Alcohol Day	*p*-Value	Control Day	Alcohol Day	*p*-Value	Control Day	Alcohol Day
STAI-T	33.1 (6.9)	30.6 (5.7)	0.527	36.7 (9.4)	36.2 (11.7)	0.796	0.505	0.217
STAI-S	26.4 (3.7)	27.0 (7.3)	0.763	31.3 (6.3)	36.8 (7.0)	0.001 *	0.020 ^‡^	0.001 ^‡^
BDI-II	3.3 (2.5)	2.1 (1.9)	0.058	8.0 (9.0)	7.4 (10.1)	0.763	0.051	0.037 ^‡^
RT-18	10.0 (4.7)	9.9 (4.5)	0.763	9.7 (4.4)	10.3 (3.8)	0.397	0.949	0.747
RT-18 F1	6.4 (2.4)	6.7 (2.2)	0.206	6.4 (2.5)	6.5 (2.5)	0.819	0.983	0.914
RT-18 F2	3.6 (2.6)	3.2 (2.8)	0.059	3.3 (2.7)	3.7 (2.4)	0.370	0.880	0.533

Mean and standard deviation (SD) are shown. Significant differences between the control day and the alcohol day (*p* < 0.05) are indicated by *. Significant differences between the hangover-resistant group and the hangover-sensitive group (*p* < 0.05) are indicated by ^‡^. Abbreviations: R = hangover-resistant group, S = hangover-sensitive group, STAI-T = state train anxiety inventory-trait, STAI-S = state train anxiety inventory-state, BDI-II = Beck’s Depression Inventory-II, RT-18 = 18-item risk-taking questionnaire, F1 = factor 1 (risk behavior), F2 = factor 2 (risk assessment).

## Data Availability

The data are available upon request from the corresponding author.

## Appendix A

**Table A1:** Overall hangover severity.

Group	Hangover-Resistant Group			Hangover-Sensitive Group			R vs. S Group, *p*-Value	
Time	Control Day	Alcohol Day	*p*-Value	Control Day	Alcohol Day	*p*-Value	Control Day	Alcohol Day
09:30	0.0 (0.0)	0.9 (1.3)	0.027	0.0 (0.0)	6.1 (2.1)	<0.001 *	1.000	<0.001 ^‡^
10:30	0.0 (0.0)	0.7 (1.2)	0.078	0.0 (0.0)	5.9 (1.8)	<0.001 *	1.000	<0.001 ^‡^
11:30	0.0 (0.0)	0.4 (0.8)	0.223	0.0 (0.0)	5.1 (2.0)	<0.001 *	1.000	<0.001 ^‡^
12:30	0.0 (0.0)	0.4 (0.9)	0.240	0.0 (0.0)	4.5 (1.8)	<0.001 *	1.000	<0.001 ^‡^
13:30	0.0 (0.0)	0.4 (0.8)	0.223	0.0 (0.0)	4.3 (2.7)	<0.001 *	1.000	<0.001 ^‡^
14:30	0.0 (0.0)	0.2 (0.4)	0.470	0.0 (0.0)	3.7 (2.3)	<0.001 *	1.000	<0.001 ^‡^
15:30	0.0 (0.0)	0.3 (0.6)	0.391	0.0 (0.0)	3.2 (2.3)	0.002 *	1.000	<0.001 ^‡^

Mean and standard deviation (SD) are shown. Significant differences (at the same timepoint) between the control day and the alcohol day (*p* < 0.0071, after Bonferroni’s correction for multiple comparisons) are indicated by *. Significant differences (at the same timepoint) between the hangover-resistant group and the hangover-sensitive group (*p* < 0.0071, after Bonferroni’s correction for multiple comparisons) are indicated by ^‡^. Abbreviations: R = hangover-resistant group, S = hangover-sensitive group.

**Table A2:** Fatigue.

Group	Hangover-Resistant Group			Hangover-Sensitive group			R vs. S Group, *p*-Value	
Time	Control Day	Alcohol Day	*p*-Value	Control Day	Alcohol Day	*p*-Value	Control Day	Alcohol Day
09:30	1.07 (1.1)	2.29 (1.7)	0.071	1.67 (1.4)	6.00 (1.8)	<0.001 *	0.298	<0.001 ^‡^
10:30	0.57 (0.8)	1.64 (1.4)	0.007 *	1.00 (1.1)	5.80 (2.2)	<0.001 *	0.238	<0.001 ^‡^
11:30	0.50 (0.8)	1.07 (0.6)	0.114	0.93 (1.0)	5.67 (2.1)	<0.001 *	0.213	<0.001 ^‡^
12:30	0.71 (0.8)	0.86 (0.9)	0.701	1.07 (1.4)	5.73 (2.7)	<0.001 *	0.759	<0.001 ^‡^
13:30	0.86 (1.0)	0.79 (1.1)	0.821	1.47 (2.1)	4.67 (2.4)	0.002 *	0.741	<0.001 ^‡^
14:30	0.79 (1.2)	0.50 (0.8)	0.701	1.13 (1.1)	4.60 (2.6)	<0.001 *	0.284	<0.001 ^‡^
15:30	0.86 (1.2)	0.64 (0.8)	0.512	1.57 (1.6)	3.80 (2.8)	0.064	0.147	<0.001 ^‡^

Mean and standard deviation (SD) are shown. Significant differences (at the same timepoint) between the control day and the alcohol day (*p* < 0.0071, after Bonferroni’s correction for multiple comparisons) are indicated by *. Significant differences (at the same timepoint) between the hangover-resistant group and the hangover-sensitive group (*p* < 0.0071, after Bonferroni’s correction for multiple comparisons) are indicated by ^‡^. Abbreviations: R = hangover-resistant group, S = hangover-sensitive group.

**Table A3:** Sleepiness.

Group	Hangover-Resistant Group			Hangover-Sensitive Group			R vs. S Group, *p*-Value	
Time	Control Day	Alcohol Day	*p*-Value	Control Day	Alcohol Day	*p*-Value	Control Day	Alcohol Day
09:30	1.29 (1.1)	2.79 (2.0)	0.119	2.13 (1.1)	6.00 (2.1)	0.004 *	0.073	0.001 ^‡^
10:30	0.86 (0.7)	1.71 (1.5)	0.109	0.87 (1.1)	6.13 (2.1)	<0.001 *	0.630	<0.001 ^‡^
11:30	1.00 (1.1)	1.29 (0.9)	0.231	0.93 (0.9)	5.67 (2.1)	<0.001 *	0.982	<0.001 ^‡^
12:30	0.86 (1.1)	1.36 (1.2)	0.206	0.67 (1.1)	5.67 (2.8)	<0.001 *	0.561	<0.001 ^‡^
13:30	0.93 (1.1)	1.07 (1.3)	0.839	1.53 (1.9)	4.33 (2.6)	0.015	0.530	<0.001 ^‡^
14:30	0.57 (1.10	0.79 (0.9)	0.572	0.40 (0.8)	4.40 (3.1)	<0.001 *	0.824	0.001 ^‡^
15:30	1.00 (1.1)	0.71 (0.9)	0.542	1.43 (2.0)	3.73 (2.8)	0.028	0.922	<0.001 ^‡^

Mean and standard deviation (SD) are shown. Significant differences (at the same timepoint) between the control day and the alcohol day (*p* < 0.0071, after Bonferroni’s correction for multiple comparisons) are indicated by *. Significant differences (at the same timepoint) between the hangover-resistant group and the hangover-sensitive group (*p* < 0.0071, after Bonferroni’s correction for multiple comparisons) are indicated by ^‡^. Abbreviations: R = hangover-resistant group, S = hangover-sensitive group.

**Table A4:** Apathy.

Group	Hangover-Resistant Group			Hangover-Sensitive Group			R vs. S Group, *p*-Value	
Time	Control Day	Alcohol Day	*p*-Value	Control Day	Alcohol Day	*p*-Value	Control Day	Alcohol Day
09:30	0.07 (0.3)	0.36 (1.1)	−	0.33 (0.6)	3.00 (2.8)	0.005 *	0.163	0.003 ^‡^
10:30	0.0 (0.0)	0.21 (0.6)	−	0.13 (0.4)	2.73 (3.0)	0.006 *	0.164	0.005 ^‡^
11:30	0.07 (0.3)	0.07 (0.3)	−	0.0 (0.0)	2.60 (2.6)	0.001 *	0.301	<0.001 ^‡^
12:30	0.14 (0.5)	0.07 (0.3)	−	0.13 (0.4)	2.67 (2.2)	<0.001 *	0.650	<0.001 ^‡^
13:30	0.0 (0.0)	0.21 (0.8)	−	0.40 (0.9)	2.47 (2.4)	0.013	0.083	<0.001 ^‡^
14:30	0.07 (0.3)	0.14 (0.4)	−	0.27 (0.8)	2.00 (2.1)	0.024	0.563	<0.001 ^‡^
15:30	0.57 (1.6)	0.36 (0.9)	−	0.14 (0.5)	1.73 (2.1)	0.019	0.308	0.005 ^‡^

Mean and standard deviation (SD) are shown. Significant differences (at the same timepoint) between the control day and the alcohol day (*p* < 0.0071, after Bonferroni’s correction for multiple comparisons) are indicated by *. For the hangover-resistant group, the main effect of control vs. alcohol day was not significant (*p* = 0.415). Therefore, no paired comparisons were conducted (indicated by −). Significant differences (at the same timepoint) between the hangover-resistant group and the hangover-sensitive group (*p* < 0.0071, after Bonferroni’s correction for multiple comparisons) are indicated by ^‡^. Abbreviations: R = hangover-resistant group, S = hangover-sensitive group.

**Table A5:** Concentration problems.

Group	Hangover-Resistant Group			Hangover-Sensitive Group			R vs. S Group, *p*-Value	
Time	Control Day	Alcohol Day	*p*-Value	Control Day	Alcohol Day	*p*-Value	Control Day	Alcohol Day
09:30	0.21 (0.4)	1.79 (2.1)	0.011	1.27 (1.3)	5.80 (2.3)	<0.001 *	0.015	<0.001 ^‡^
10:30	0.29 (0.8)	1.29 (2.4)	0.009	1.00 (1.4)	5.27 (2.7)	<0.001 *	0.070	<0.001 ^‡^
11:30	0.36 (0.6)	0.93 (1.0)	0.130	1.00 (1.1)	4.53 (2.3)	<0.001 *	0.105	<0.001 ^‡^
12:30	0.29 (0.8)	0.64 (1.2)	0.354	1.13 (1.1)	4.93 (3.0)	<0.001 *	0.008	<0.001 ^‡^
13:30	0.29 (0.6)	0.57 (0.9)	0.498	1.87 (2.3)	4.00 (2.8)	0.015	0.024	<0.001 ^‡^
14:30	0.21 (0.4)	0.36 (0.6)	0.821	1.27 (1.6)	4.13 (2.7)	0.003 *	0.039	<0.001 ^‡^
15:30	0.43 (0.8)	0.43 (0.8)	0.946	1.71 (2.1)	3.40 (2.8)	0.055	0.066	<0.001 ^‡^

Mean and standard deviation (SD) are shown. Significant differences (at the same timepoint) between the control day and the alcohol day (*p* < 0.0071, after Bonferroni’s correction for multiple comparisons) are indicated by *. Significant differences (at the same timepoint) between the hangover-resistant group and the hangover-sensitive group (*p* < 0.0071, after Bonferroni’s correction for multiple comparisons) are indicated by ^‡^. Abbreviations: R = hangover-resistant group, S = hangover-sensitive group.

**Table A6:** Weakness.

Group	Hangover-Resistant Group			Hangover-Sensitive Group			R vs. S Group, *p*-Value	
Time	Control Day	Alcohol Day	*p*-Value	Control Day	Alcohol Day	*p*-Value	Control Day	Alcohol Day
09:30	0.14 (0.4)	0.79 (1.2)	0.130	0.13 (0.4)	3.80 (3.1)	<0.001 *	0.942	0.002 ^‡^
10:30	0.0 (0.0)	0.50 (0.9)	0.161	0.07 (0.3)	3.40 (3.0)	<0.001 *	0.334	0.001 ^‡^
11:30	0.0 (0.0)	0.14 (0.4)	0.588	0.07 (0.3)	2.67 (2.8)	0.004 *	0.334	<0.001 ^‡^
12:30	0.0 (0.0)	0.14 (0.4)	0.557	0.07 (0.3)	2.67 (2.9)	0.004 *	0.334	0.002 ^‡^
13:30	0.0 (0.0)	0.07 (0.3)	0.786	0.13 (0.4)	2.53 (2.7)	0.004 *	0.164	<0.001 ^‡^
14:30	0.21 (0.8)	0.21 (0.4)	0.668	0.0 (0.0)	2.40 (2.4)	0.002 *	0.301	0.001 ^‡^
15:30	0.0 (0.0)	0.14 (0.4)	0.557	0.07 (0.3)	1.87 (2.6)	0.040	0.317	0.008

Mean and standard deviation (SD) are shown. Significant differences (at the same timepoint) between the control day and the alcohol day (*p* < 0.0071, after Bonferroni’s correction for multiple comparisons) are indicated by *. Significant differences (at the same timepoint) between the hangover-resistant group and the hangover-sensitive group (*p* < 0.0071, after Bonferroni’s correction for multiple comparisons) are indicated by ^‡^. Abbreviations: R = hangover-resistant group, S = hangover-sensitive group.

**Table A7:** Clumsiness.

Group	Hangover-Resistant Group			Hangover-Sensitive Group			R vs. S Group, *p*-Value	
Time	Control Day	Alcohol Day	*p*-Value	Control Day	Alcohol Day	*p*-Value	Control Day	Alcohol Day
09:30	0.29 (0.6)	0.71 (1.1)	0.416	0.20 (0.4)	4.20 (3.0)	<0.001 *	0.853	<0.001 ^‡^
10:30	0.07 (0.3)	0.29 (0.6)	0.557	0.20 (0.4)	3.60 (3.2)	<0.001 *	0.324	<0.001 ^‡^
11:30	0.0 (0.0)	0.14 (0.5)	0.769	0.13 (0.4)	2.87 (3.2)	0.003 *	0.164	<0.001 ^‡^
12:30	0.0 (0.0)	0.21 (0.8)	0.668	0.20 (0.6)	2.80 (3.1)	0.004 *	0.164	<0.001 ^‡^
13:30	0.0 (0.0)	0.07 (0.3)	0.857	0.0 (0.0)	2.73 (3.2)	<0.001 *	1.000	<0.001 ^‡^
14:30	0.0 (0.0)	0.21 (0.8)	0.668	0.0 (0.0)	2.60 (3.1)	<0.001 *	1.000	<0.001 ^‡^
15:30	0.07 (0.3)	0.14 (0.5)	0.910	0.07 (0.3)	2.20 (3.1)	0.008	1.000	<0.001 ^‡^

Mean and standard deviation (SD) are shown. Significant differences (at the same timepoint) between the control day and the alcohol day (*p* < 0.0071, after Bonferroni’s correction for multiple comparisons) are indicated by *. Significant differences (at the same timepoint) between the hangover-resistant group and the hangover-sensitive group (*p* < 0.0071, after Bonferroni’s correction for multiple comparisons) are indicated by ^‡^. Abbreviations: R = hangover-resistant group, S = hangover-sensitive group.

**Table A8:** Confusion.

Group	Hangover-Resistant Group			Hangover-Sensitive Group			R vs. S Group, *p*-Value	
Time	Control Day	Alcohol Day	*p*-Value	Control Day	Alcohol Day	*p*-Value	Control Day	Alcohol Day
09:30	0.0 (0.0)	0.21 (0.6)	−	0.0 (0.0)	2.07 (2.9)	0.055	1.000	0.072
10:30	0.0 (0.0)	0.29 (0.8)	−	0.0 (0.0)	2.13 (3.1)	0.055	1.000	0.086
11:30	0.0 (0.0)	0.07 (0.3)	−	0.0 (0.0)	1.87 (2.9)	0.074	1.000	0.029
12:30	0.0 (0.0)	0.0 (0.0)	−	0.13 (0.4)	2.13 (3.2)	0.052	0.164	0.004 ^‡^
13:30	0.0 (0.0)	0.07 (0.3)	−	0.0 (0.0)	1.33 (2.2)	0.175	1.000	0.034
14:30	0.0 (0.0)	0.0 (0.0)	−	0.07 (0.3)	1.20 (2.1)	0.366	0.334	0.020
15:30	0.0 (0.0)	0.07 (0.3)	−	0.0 (0.0)	1.07 (2.0)	0.354	1.000	0.136

Mean and standard deviation (SD) are shown. No significant differences (at the same timepoint) between the control day and the alcohol day (*p* < 0.0071, after Bonferroni’s correction for multiple comparisons) were found. For the hangover-resistant group, the main effect of control vs. alcohol day was not significant (*p* = 0.161). Therefore, no paired comparisons were conducted (indicated by −). Significant differences (at the same timepoint) between the hangover-resistant group and the hangover-sensitive group (*p* < 0.0071, after Bonferroni’s correction for multiple comparisons) are indicated by ^‡^. Abbreviations: R = hangover-resistant group, S = hangover-sensitive group.

**Table A9:** Headache.

Group	Hangover-Resistant Group			Hangover-Sensitive Group			R vs. S Group, *p*-Value	
Time	Control Day	Alcohol Day	*p*-Value	Control Day	Alcohol Day	*p*-Value	Control Day	Alcohol Day
09:30	0.21 (0.6)	0.64 (1.2)	−	0.53 (0.7)	4.40 (2.7)	<0.001 *	0.148	<0.001 ^‡^
10:30	0.21 (0.6)	0.43 (0.6)	−	0.60 (0.7)	4.47 (3.1)	<0.001 *	0.081	<0.001 ^‡^
11:30	0.21 (0.6)	0.36 (0.5)	−	0.47 (0.9)	4.00 (2.6)	<0.001 *	0.405	<0.001 ^‡^
12:30	0.21 (0.6)	0.14 (0.4)	−	0.67 (1.0)	3.07 (2.3)	0.010	0.127	<0.001 ^‡^
13:30	0.21 (0.6)	0.36 (0.5)	−	0.67 (1.0)	3.07 (2.2)	0.007 *	0.127	<0.001 ^‡^
14:30	0.14 (0.5)	0.29 (0.5)	−	0.80 (1.3)	2.53 (2.2)	0.155	0.028	0.003 ^‡^
15:30	0.29 (1.1)	0.36 (1.1)	−	0.57 (1.1)	2.13 (2.1)	0.136	0.191	0.002 ^‡^

Mean and standard deviation (SD) are shown. Significant differences (at the same timepoint) between the control day and the alcohol day (*p* < 0.0071, after Bonferroni’s correction for multiple comparisons) are indicated by *. For the hangover-resistant group, the main effect of control vs. alcohol day was not significant (*p* = 0.404). Therefore, no paired comparisons were conducted (indicated by −). Significant differences (at the same timepoint) between the hangover-resistant group and the hangover-sensitive group (*p* < 0.0071, after Bonferroni’s correction for multiple comparisons) are indicated by ^‡^. Abbreviations: R = hangover-resistant group, S = hangover-sensitive group.

**Table A10:** Stomach pain.

Group	Hangover-Resistant Group			Hangover-Sensitive Group			R vs. S Group, *p*-Value	
Time	Control Day	Alcohol Day	*p*-Value	Control Day	Alcohol Day	*p*-Value	Control Day	Alcohol Day
09:30	0.07 (0.3)	0.21 (0.6)	−	0.33 (0.8)	3.07 (3.3)	0.013	0.308	0.005 ^‡^
10:30	0.0 (0.0)	0.14 (0.4)	−	0.20 (0.4)	2.73 (2.9)	0.009	0.082	0.002 ^‡^
11:30	0.0 (0.0)	0.0 (0.0)	−	0.07 (0.3)	2.27 (2.5)	0.017	0.334	<0.001 ^‡^
12:30	0.0 (0.0)	0.0 (0.0)	−	0.13 (0.5)	1.53 (1.8)	0.086	0.334	<0.001 ^‡^
13:30	0.0 (0.0)	0.0 (0.0)	−	0.07 (0.3)	2.53 (3.1)	0.023	0.334	<0.001 ^‡^
14:30	0.0 (0.0)	0.07 (0.3)	−	0.0 (0.0)	2.13 (3.4)	0.071	1.000	0.064
15:30	0.07 (0.3)	0.0 (0.0)	−	0.0 (0.0)	2.27 (3.1)	0.016	0.317	0.004 ^‡^

Mean and standard deviation (SD) are shown. No significant differences (at the same timepoint) between the control day and the alcohol day (*p* < 0.0071, after Bonferroni’s correction for multiple comparisons) were found. For the hangover-resistant group, the main effect of control vs. alcohol day was not significant (*p* = 0.123). Therefore, no paired comparisons were conducted (indicated by −). Significant differences (at the same timepoint) between the hangover-resistant group and the hangover-sensitive group (*p* < 0.0071, after Bonferroni’s correction for multiple comparisons) are indicated by ^‡^. Abbreviations: R = hangover-resistant group, S = hangover-sensitive group.

**Table A11:** Nausea.

Group	Hangover-Resistant Group			Hangover-Sensitive Group			R vs. S Group, *p*-Value	
Time	Control Day	Alcohol Day	*p*-Value	Control Day	Alcohol Day	*p*-Value	Control Day	Alcohol Day
09:30	0.07 (0.3)	1.14 (1.7)	0.130	0.13 (0.4)	4.47 (3.3)	<0.001 *	0.591	0.002 ^‡^
10:30	0.0 (0.0)	0.43 (0.6)	0.142	0.0 (0.0)	4.33 (2.7)	<0.001 *	1.000	<0.001 ^‡^
11:30	0.0 (0.0)	0.14 (0.4)	0.557	0.0 (0.0)	2.87 (2.2)	0.002 *	1.000	<0.001 ^‡^
12:30	0.0 (0.0)	0.29 (0.6)	0.320	0.0 (0.0)	2.53 (2.2)	0.011	1.000	0.004 ^‡^
13:30	0.07 (0.3)	0.14 (0.4)	0.786	0.0 (0.0)	3.20 (3.6)	0.001 *	0.301	<0.001 ^‡^
14:30	0.0 (0.0)	0.21 (0.4)	0.378	0.0 (0.0)	3.07 (3.2)	<0.001 *	1.000	0.003 ^‡^
15:30	0.21 (0.8)	0.14 (0.4)	0.769	0.0 (0.0)	2.60 (3.2)	0.007 *	0.317	0.005 ^‡^

Mean and standard deviation (SD) are shown. Significant differences (at the same timepoint) between the control day and the alcohol day (*p* < 0.0071, after Bonferroni’s correction for multiple comparisons) are indicated by *. Significant differences (at the same timepoint) between the hangover-resistant group and the hangover-sensitive group (*p* < 0.0071, after Bonferroni’s correction for multiple comparisons) are indicated by ^‡^. Abbreviations: R = hangover-resistant group, S = hangover-sensitive group.

**Table A12:** Vomiting.

Group	Hangover-Resistant Group			Hangover-Sensitive Group			R vs. S Group, *p*-Value	
Time	Control Day	Alcohol Day	*p*-Value	Control Day	Alcohol Day	*p*-Value	Control Day	Alcohol Day
09:30	0.07 (0.3)	0.21 (0.6)	−	0.0 (0.0)	1.27 (2.2)	0.090	0.301	0.181
10:30	0.0 (0.0)	0.14 (0.4)	−	0.0 (0.0)	1.07 (2.0)	0.109	1.000	0.180
11:30	0.0 (0.0)	0.14 (0.4)	−	0.0 (0.0)	0.80 (2.0)	0.320	1.000	0.595
12:30	0.0 (0.0)	0.0 (0.0)	−	0.0 (0.0)	0.40 (0.9)	0.331	1.000	0.083
13:30	0.07 (0.3)	0.07 (0.3)	−	0.0 (0.0)	0.87 (1.7)	0.206	0.301	0.136
14:30	0.0 (0.0)	0.07 (0.3)	−	0.0 (0.0)	0.87 (1.8)	0.190	1.000	0.145
15:30	0.0 (0.0)	0.0 (0.0)	−	0.0 (0.0)	0.33 (0.7)	0.484	1.000	0.083

Mean and standard deviation (SD) are shown. No significant differences (at the same timepoint) between the control day and the alcohol day (*p* < 0.0071, after Bonferroni’s correction for multiple comparisons) were found. For the hangover-resistant group, the main effect of control vs. alcohol day was not significant (*p* = 0.131). Therefore, no paired comparisons were conducted (indicated by −). No significant differences (at the same timepoint) between the hangover-resistant group and the hangover-sensitive group (*p* < 0.0071, after Bonferroni’s correction for multiple comparisons) were found. Abbreviations: R = hangover-resistant group, S = hangover-sensitive group.

**Table A13:** Shivering.

Group	Hangover-Resistant Group			Hangover-Sensitive Group			R vs. S Group, *p*-Value	
Time	Control Day	Alcohol Day	*p*-Value	Control Day	Alcohol Day	*p*-Value	Control Day	Alcohol Day
09:30	0.0 (0.0)	0.36 (1.1)	−	0.07 (0.3)	2.40 (3.1)	0.006 *	0.334	0.011
10:30	0.0 (0.0)	0.14 (0.4)	−	0.07 (0.3)	2.27 (2.9)	0.013	0.334	0.007 ^‡^
11:30	0.0 (0.0)	0.07 (0.3)	−	0.07 (0.3)	2.13 (2.4)	0.002 *	0.334	<0.001 ^‡^
12:30	0.0 (0.0)	0.07 (0.3)	−	0.20 (0.6)	2.07 (2.1)	0.014	0.164	<0.001 ^‡^
13:30	0.0 (0.0)	0.14 (0.4)	−	0.13 (0.4)	1.60 (1.8)	0.078	0.164	0.007 ^‡^
14:30	0.0 (0.0)	0.07 (0.3)	−	0.07 (0.3)	1.53 (2.1)	0.082	0.334	0.007 ^‡^
15:30	0.0 (0.0)	0.07 (0.3)	−	0.0 (0.0)	1.33 (2.2)	0.119	1.000	0.017

Mean and standard deviation (SD) are shown. Significant differences (at the same timepoint) between the control day and the alcohol day (*p* < 0.0071, after Bonferroni’s correction for multiple comparisons) are indicated by *. For the hangover-resistant group, the main effect of control vs. alcohol day was not significant (*p* = 0.102). Therefore, no paired comparisons were conducted (indicated by −). Significant differences (at the same timepoint) between the hangover-resistant group and the hangover-sensitive group (*p* < 0.0071, after Bonferroni’s correction for multiple comparisons) are indicated by ^‡^. Abbreviations: R = hangover-resistant group, S = hangover-sensitive group.

**Table A14:** Dizziness.

Group	Hangover-Resistant Group			Hangover-Sensitive Group			R vs. S Group, *p*-Value	
Time	Control Day	Alcohol Day	*p*-Value	Control Day	Alcohol Day	*p*-Value	Control Day	Alcohol Day
09:30	0.07 (0.3)	0.86 (1.5)	0.155	0.0 (0.0)	3.13 (3.0)	<0.001 *	0.301	0.019
10:30	0.07 (0.3)	0.36 (0.9)	0.668	0.0 (0.0)	2.87 (2.7)	<0.001 *	0.301	<0.001 ^‡^
11:30	0.07 (0.3)	0.21 (0.4)	0.588	0.0 (0.0)	2.20 (2.2)	0.005 *	0.301	0.003 ^‡^
12:30	0.0 (0.0)	0.21 (0.4)	0.391	0.0 (0.0)	2.40 (2.4)	0.002 *	1.000	0.003 ^‡^
13:30	0.0 (0.0)	0.07 (0.3)	0.752	0.0 (0.0)	1.67 (2.0)	0.018	1.000	0.003 ^‡^
14:30	0.07 (0.3)	0.14 (0.4)	0.786	0.0 (0.0)	1.20 (1.5)	0.042	0.301	0.017
15:30	0.07 (0.3)	0.0 (0.0)	0.786	0.0 (0.0)	1.33 (2.2)	0.044	0.317	0.004 ^‡^

Mean and standard deviation (SD) are shown. Significant differences (at the same timepoint) between the control day and the alcohol day (*p* < 0.0071, after Bonferroni’s correction for multiple comparisons) are indicated by *. Significant differences (at the same timepoint) between the hangover-resistant group and the hangover-sensitive group (*p* < 0.0071, after Bonferroni’s correction for multiple comparisons) are indicated by ^‡^. Abbreviations: R = hangover-resistant group, S = hangover-sensitive group.

**Table A15:** Sensitivity to light.

Group	Hangover-Resistant Group			Hangover-Sensitive Group			R vs. S Group, *p*-Value	
Time	Control Day	Alcohol Day	*p*-Value	Control Day	Alcohol Day	*p*-Value	Control Day	Alcohol Day
09:30	0.14 (0.4)	0.21 (0.6)	−	0.33 (1.0)	2.87 (2.6)	0.001 *	1.000	<0.001 ^‡^
10:30	0.0 (0.0)	0.14 (0.4)	−	0.33 (1.0)	2.67 (2.7)	<0.001 *	0.164	<0.001 ^‡^
11:30	0.0 (0.0)	0.14 (0.4)	−	0.33 (1.0)	2.13 (2.4)	0.003 *	0.164	<0.001 ^‡^
12:30	0.0 (0.0)	0.07 (0.3)	−	0.27 (1.0)	1.27 (1.6)	0.104	0.334	0.006 ^‡^
13:30	0.0 (0.0)	0.14 (0.5)	−	0.27 (1.0)	1.60 (2.1)	0.021	0.334	0.016
14:30	0.0 (0.0)	0.07 (0.3)	−	0.27 (1.0)	1.53 (1.9)	0.024	0.334	0.013
15:30	0.0 (0.0)	0.14 (0.5)	−	0.0 (0.0)	1.13 (1.4)	0.055	1.000	0.018

Mean and standard deviation (SD) are shown. Significant differences (at the same timepoint) between the control day and the alcohol day (*p* < 0.0071, after Bonferroni’s correction for multiple comparisons) are indicated by *. For the hangover-resistant group, the main effect of control vs. alcohol day was not significant (*p* = 0.102). Therefore, no paired comparisons were conducted (indicated by −). Significant differences (at the same timepoint) between the hangover-resistant group and hangover-sensitive group (*p* < 0.0071, after Bonferroni’s correction for multiple comparisons) are indicated by ^‡^. Abbreviations: R = hangover-resistant group, S = hangover-sensitive group.

**Table A16:** Sensitivity to sound.

Group	Hangover-Resistant Group			Hangover-Sensitive Group			R vs. S Group, *p*-Value	
Time	Control Day	Alcohol Day	*p*-Value	Control Day	Alcohol Day	*p*-Value	Control Day	Alcohol Day
09:30	0.07 (0.3)	0.36 (0.6)	0.343	0.07 (0.3)	2.40 (2.9)	0.017	0.960	0.056
10:30	0.0 (0.0)	0.14 (0.4)	0.572	0.0 (0.0)	2.07 (2.6)	0.008	1.000	0.013
11:30	0.0 (0.0)	0.14 (0.4)	0.572	0.0 (0.0)	1.53 (2.4)	0.033	1.000	0.042
12:30	0.0 (0.0)	0.07 (0.3)	0.786	0.0 (0.0)	0.93 (1.6)	0.271	1.000	0.073
13:30	0.0 (0.0)	0.21 (0.8)	0.651	0.0 (0.0)	0.93 (1.8)	0.373	1.000	0.154
14:30	0.0 (0.0)	0.07 (0.3)	0.786	0.0 (0.0)	0.50 (1.3)	0.251	1.000	0.122
15:30	0.0 (0.0)	0.21 (0.6)	0.470	0.29 (1.1)	0.60 (1.1)	0.606	0.317	0.355

Mean and standard deviation (SD) are shown. No significant differences (at the same timepoint) between the control day and the alcohol day (*p* < 0.0071, after Bonferroni’s correction for multiple comparisons) were found. No significant differences (at the same timepoint) between the hangover-resistant group and the hangover-sensitive group (*p* < 0.0071, after Bonferroni’s correction for multiple comparisons) were found. Abbreviations: R = hangover-resistant group, S = hangover-sensitive group.

**Table A17:** Sweating.

Group	Hangover-Resistant Group			Hangover-Sensitive Group			R vs. S Group, *p*-Value	
Time	Control Day	Alcohol Day	*p*-Value	Control Day	Alcohol Day	*p*-Value	Control Day	Alcohol Day
09:30	0.0 (0.0)	0.07 (0.3)	−	0.07 (0.3)	1.27 (2.6)	0.223	0.334	0.074
10:30	0.0 (0.0)	0.07 (0.3)	−	0.0 (0.0)	1.13 (2.1)	0.136	1.000	0.074
11:30	0.07 (0.3)	0.0 (0.0)	−	0.0 (0.0)	0.73 (1.7)	0.331	0.301	0.041
12:30	0.07 (0.3)	0.07 (1.3)	−	0.0 (0.0)	0.53 (1.2)	0.542	0.301	0.291
13:30	0.07 (0.3)	0.0 (0.0)	−	0.0 (0.0)	0.47 (1.4)	0.572	0.301	0.164
14:30	0.07 (0.3)	0.07 (0.3)	−	0.0 (0.0)	0.27 (0.7)	0.651	0.301	0.536
15:30	0.07 (0.3)	0.14 (0.5)	−	0.0 (0.0)	0.0 (0.6)	0.701	0.317	0.620

Mean and standard deviation (SD) are shown. No significant differences (at the same timepoint) between the control day and the alcohol day (*p* < 0.0071, after Bonferroni’s correction for multiple comparisons) were found. For the hangover-resistant group, the main effect of control vs. alcohol day was not significant (*p* = 0.873). Therefore, no paired comparisons were conducted (indicated by −). No significant differences (at the same timepoint) between the hangover-resistant group and the hangover-sensitive group (*p* < 0.0071, after Bonferroni’s correction for multiple comparisons) were found. Abbreviations: R = hangover-resistant group, S = hangover-sensitive group.

**Table A18:** Heart pounding.

Group	Hangover-Resistant Group			Hangover-Sensitive Group			R vs. S Group, *p*-Value	
Time	Control Day	Alcohol Day	*p*-Value	Control Day	Alcohol Day	*p*-Value	Control Day	Alcohol Day
09:30	0.0 (0.0)	0.21 (0.8)	−	0.20 (0.6)	1.53 (2.3)	0.114	0.164	0.021
10:30	0.0 (0.0)	0.07 (0.3)	−	0.0 (0.0)	1.27 (2.2)	0.090	1.000	0.034
11:30	0.0 (0.0)	0.0 (0.0)	−	0.0 (0.0)	0.93 (1.9)	0.320	1.000	0.041
12:30	0.0 (0.0)	0.07 (0.3)	−	0.0 (0.0)	0.40 (1.1)	0.603	1.000	0.536
13:30	0.0 (0.0)	0.0 (0.0)	−	0.0 (0.0)	0.47 (1.2)	0.557	1.000	0.164
14:30	0.0 (0.0)	0.07 (0.3)	−	0.07 (0.3)	0.40 (1.1)	0.857	0.334	0.536
15:30	0.0 (0.0)	0.07 (0.3)	−	0.0 (0.0)	0.40 (1.3)	0.619	1.000	0.563

Mean and standard deviation (SD) are shown. No significant differences (at the same timepoint) between the control day and the alcohol day (*p* < 0.0071, after Bonferroni’s correction for multiple comparisons) were found. For the hangover-resistant group, the main effect of control vs. alcohol day was not significant (*p* = 0.448). Therefore, no paired comparisons were conducted (indicated by −). No significant differences (at the same timepoint) between the hangover-resistant group and the hangover-sensitive group (*p* < 0.0071, after Bonferroni’s correction for multiple comparisons) were found. Abbreviations: R = hangover-resistant group, S = hangover-sensitive group.

**Table A19:** Heart racing.

Group	Hangover-Resistant Group			Hangover-Sensitive Group			R vs. S Group, *p*-Value	
Time	Control Day	Alcohol Day	*p*-Value	Control Day	Alcohol Day	*p*-Value	Control Day	Alcohol Day
09:30	0.0 (0.0)	0.14 (0.5)	−	0.07 (0.3)	1.27 (2.2)	0.240	0.334	0.074
10:30	0.0 (0.0)	0.07 (0.3)	−	0.0 (0.0)	1.07 (2.0)	0.299	1.000	0.136
11:30	0.0 (0.0)	0.07 (0.3)	−	0.0 (0.0)	0.87 (1.9)	0.309	1.000	0.145
12:30	0.0 (0.0)	0.07 (0.3)	−	0.0 (0.0)	0.33 (0.9)	0.588	1.000	0.536
13:30	0.0 (0.0)	0.07 (0.3)	−	0.0 (0.0)	0.47 (1.2)	0.456	1.000	0.536
14:30	0.0 (0.0)	0.07 (0.3)	−	0.0 (0.0)	0.20 (0.6)	0.684	1.000	0.563
15:30	0.0 (0.0)	0.0 (0.0)	−	0.0 (0.0)	0.27 (0.7)	0.651	1.000	0.164

Mean and standard deviation (SD) are shown. No significant differences (at the same timepoint) between the control day and the alcohol day (*p* < 0.0071, after Bonferroni’s correction for multiple comparisons) were found. For the hangover-resistant group, the main effect of control vs. alcohol day was not significant (*p* = 0.448). Therefore, no paired comparisons were conducted (indicated by −). No significant differences (at the same timepoint) between the hangover-resistant group and the hangover-sensitive group (*p* < 0.0071, after Bonferroni’s correction for multiple comparisons) were found. Abbreviations: R = hangover-resistant group, S = hangover-sensitive group.

**Table A20:** Thirst.

Group	Hangover-Resistant Group			Hangover-Sensitive Group			R vs. S Group, *p*-Value	
Time	Control Day	Alcohol Day	*p*-Value	Control Day	Alcohol Day	*p*-Value	Control Day	Alcohol Day
09:30	1.29 (1.5)	1.86 (1.9)	0.416	1.67 (1.4)	5.33 (2.6)	<0.001 *	0.471	0.002 ^‡^
10:30	0.86 (1.1)	1.79 (1.8)	0.299	1.73 (2.3)	4.73 (2.7)	<0.001 *	0.368	0.004 ^‡^
11:30	0.14 (0.4)	0.36 (0.5)	0.542	1.20 (2.0)	3.00 (2.5)	0.014	0.095	0.003 ^‡^
12:30	0.36 (0.6)	0.57 (0.9)	0.668	1.27 (2.4)	2.67 (2.7)	0.032	0.593	0.020
13:30	0.36 (0.7)	0.29 (0.6)	0.874	1.00 (1.6)	2.40 (2.6)	0.047	0.230	0.006 ^‡^
14:30	0.36 (0.8)	0.29 (0.6)	0.804	1.27 (1.9)	2.13 (2.3)	0.125	0.192	0.006 ^‡^
15:30	0.50 (1.0)	0.29 (0.8)	0.470	1.36 (2.2)	1.93 (2.7)	0.331	0.300	0.013

Mean and standard deviation (SD) are shown. Significant differences (at the same timepoint) between the control day and the alcohol day (*p* < 0.0071, after Bonferroni’s correction for multiple comparisons) are indicated by *. Significant differences (at the same timepoint) between the hangover-resistant group and the hangover-sensitive group (*p* < 0.0071, after Bonferroni’s correction for multiple comparisons) are indicated by ^‡^. Abbreviations: R = hangover-resistant group, S = hangover-sensitive group.

**Table A21:** Regret.

Group	Hangover-Resistant Group			Hangover-Sensitive Group			R vs. S Group, *p*-Value	
Time	Control Day	Alcohol Day	*p*-Value	Control Day	Alcohol Day	*p*-Value	Control Day	Alcohol Day
09:30	0.0 (0.0)	0.57 (1.9)	−	0.0 (0.0)	1.87 (3.2)	0.130	1.000	0.233
10:30	0.0 (0.0)	0.21 (0.8)	−	0.0 (0.0)	1.93 (2.6)	0.058	1.000	0.019
11:30	0.0 (0.0)	0.36 (1.3)	−	0.0 (0.0)	1.33 (2.6)	0.331	1.000	0.154
12:30	0.0 (0.0)	0.43 (1.6)	−	0.0 (0.0)	1.53 (2.4)	0.071	1.000	0.055
13:30	0.0 (0.0)	0.36 (1.3)	−	0.0 (0.0)	1.07 (2.1)	0.240	1.000	0.102
14:30	0.0 (0.0)	0.29 (1.1)	−	0.0 (0.0)	0.87 (1.8)	0.331	1.000	0.102
15:30	0.0 (0.0)	0.21 (0.8)	−	0.0 (0.0)	0.93 (1.9)	0.249	1.000	0.102

Mean and standard deviation (SD) are shown. No significant differences (at the same timepoint) between the control day and the alcohol day (*p* < 0.0071, after Bonferroni’s correction for multiple comparisons) were found. For the hangover-resistant group, the main effect of control vs. alcohol day was not significant (*p* = 0.163). Therefore, no paired comparisons were conducted (indicated by −). No significant differences (at the same timepoint) between the hangover-resistant group and the hangover-sensitive group (*p* < 0.0071, after Bonferroni’s correction for multiple comparisons) were found. Abbreviations: R = hangover-resistant group, S = hangover-sensitive group.

**Table A22:** Anxiety.

Group.	Hangover-Resistant Group			Hangover-Sensitive Group			R vs. S Group, *p*-Value	
Time	Control Day	Alcohol Day	*p*-Value	Control Day	Alcohol Day	*p*-Value	Control Day	Alcohol Day
09:30	0.0 (0.0)	0.21 (0.8)	−	0.0 (0.0)	1.20 (2.4)	0.183	1.000	0.154
10:30	0.0 (0.0)	0.21 (0.8)	−	0.0 (0.0)	0.87 (1.8)	0.268	1.000	0.174
11:30	0.0 (0.0)	0.14 (0.5)	−	0.0 (0.0)	0.67 (1.6)	0.288	1.000	0.308
12:30	0.0 (0.0)	0.29 (1.1)	−	0.0 (0.0)	0.40 (0.7)	0.259	1.000	0.220
13:30	0.0 (0.0)	0.29 (1.1)	−	0.0 (0.0)	0.40 (0.9)	0.366	1.000	0.382
14:30	0.0 (0.0)	0.14 (0.5)	−	0.20 (0.8)	0.27 (0.6)	0.718	0.334	0.363
15:30	0.0 (0.0)	0.21 (0.8)	−	0.0 (0.0)	0.20 (0.6)	0.668	1.000	0.650

Mean and standard deviation (SD) are shown. No significant differences (at the same timepoint) between the control day and the alcohol day (*p* < 0.0071, after Bonferroni’s correction for multiple comparisons) were found. For the hangover-resistant group, the main effect of control vs. alcohol day was not significant (*p* = 0.448). Therefore, no paired comparisons were conducted (indicated by −). No significant differences (at the same timepoint) between the hangover-resistant group and the hangover-sensitive group (*p* < 0.0071, after Bonferroni’s correction for multiple comparisons) were found. Abbreviations: R = hangover-resistant group, S = hangover-sensitive group.

**Table A23:** Depression.

Group	Hangover-Resistant Group			Hangover-Sensitive Group			R vs. S Group, *p*-Value	
Time	Control Day	Alcohol Day	*p*-Value	Control Day	Alcohol Day	*p*-Value	Control Day	Alcohol Day
09:30	0.0 (0.0)	0.43 (1.6)	−	0.0 (0.0)	1.60 (3.0)	0.095	1.000	0.090
10:30	0.0 (0.0)	0.43 (1.6)	−	0.07 (0.3)	1.07 (2.2)	0.231	0.334	0.196
11:30	0.0 (0.0)	0.36 (1.3)	−	0.0 (0.0)	1.07 (2.3)	0.136	1.000	0.185
12:30	0.0 (0.0)	0.36 (1.3)	−	0.0 (0.0)	0.80 (1.8)	0.259	1.000	0.196
13:30	0.0 (0.0)	0.36 (1.3)	−	0.0 (0.0)	0.73 (1.8)	0.416	1.000	0.344
14:30	0.0 (0.0)	0.29 (1.1)	−	0.13 (0.5)	0.67 (1.6)	0.470	0.334	0.196
15:30	0.0 (0.0)	0.29 (1.1)	−	0.14 (0.5)	0.53 (1.8)	0.839	0.317	0.592

Mean and standard deviation (SD) are shown. No significant differences (at the same timepoint) between the control day and the alcohol day (*p* < 0.0071, after Bonferroni’s correction for multiple comparisons) were found. For the hangover-resistant group, the main effect of control vs. alcohol day was not significant (*p* = 0.448). Therefore, no paired comparisons were conducted (indicated by −). No significant differences (at the same timepoint) between the hangover-resistant group and the hangover-sensitive group (*p* < 0.0071, after Bonferroni’s correction for multiple comparisons) were found. Abbreviations: R = hangover-resistant group, S = hangover-sensitive group.

**Table A24:** Reduced appetite.

Group	Hangover-Resistant Group			Hangover-Sensitive Group			R vs. S Group, *p*-Value	
Time	Control Day	Alcohol Day	*p*-Value	Control Day	Alcohol Day	*p*-Value	Control Day	Alcohol Day
09:30	0.07 (0.3)	0.64 (1.6)	−	0.40 (0.7)	4.33 (2.9)	<0.001 *	0.154	<0.001 ^‡^
10:30	0.07 (0.3)	0.57 (2.1)	−	0.27 (0.6)	3.73 (3.3)	0.004 *	0.308	0.003 ^‡^
11:30	0.07 (0.3)	0.43 (1.6)	−	0.20 (0.4)	2.80 (3.3)	0.023	0.324	0.003 ^‡^
12:30	0.0 (0.0)	0.36 (1.3)	−	0.0 (0.0)	2.53 (3.0)	0.004 *	1.000	0.005 ^‡^
13:30	0.21 (0.8)	0.50 (1.9)	−	0.07 (0.3)	2.47 (3.4)	0.025	0.921	0.027
14:30	0.0 (0.0)	0.29 (1.1)	−	0.0 (0.0)	2.53 (3.3)	0.005 *	1.000	0.005 ^‡^
15:30	0.0 (0.0)	0.36 (1.3)	−	0.0 (0.0)	2.13 (3.2)	0.042	1.000	0.027

Mean and standard deviation (SD) are shown. Significant differences (at the same timepoint) between the control day and the alcohol day (*p* < 0.0071, after Bonferroni’s correction for multiple comparisons) are indicated by *. For the hangover-resistant group, the main effect of control vs. alcohol day was not significant (*p* = 0.065). Therefore, no paired comparisons were conducted (indicated by −). Significant differences (at the same timepoint) between the hangover-resistant group and the hangover-sensitive group (*p* < 0.0071, after Bonferroni’s correction for multiple comparisons) are indicated by ^‡^. Abbreviations: R = hangover-resistant group, S = hangover-sensitive group.

**Table A25:** Karolinska Sleepiness Scale.

Group	Hangover-Resistant Group			Hangover-Sensitive Group			R vs. S Group, *p*-Value	
Time	Control Day	Alcohol Day	*p*-Value	Control Day	Alcohol Day	*p*-Value	Control Day	Alcohol Day
09:30	3.4 (1.2)	5.0 (1.7)	0.009	3.9 (1.5)	7.1 (1.3)	<0.001 *	0.474	0.002 ^‡^
10:30	3.1 (1.1)	5.1 (1.7)	<0.001 *	3.6 (1.5)	7.3 (1.2)	<0.001 *	0.492	0.001 ^‡^
11:30	3.3 (1.1)	4.1 (1.2)	0.021	3.4 (1.1)	7.1 (1.2)	<0.001 *	0.695	<0.001 ^‡^
12:30	3.1 (0.9)	4.3 (1.8)	0.019	3.9 (1.6)	7.5 (1.4)	<0.001 *	0.184	<0.001 ^‡^
13:30	3.4 (1.7)	4.1 (1.8)	0.136	4.3 (1.9)	6.5 (1.6)	0.032	0.140	0.001 ^‡^
14:30	3.2 (1.4)	4.1 (1.5)	0.067	3.6 (1.3)	6.8 (1.6)	<0.001 *	0.275	<0.001 ^‡^
15:30	3.4 (1.3)	3.5 (1.3)	0.928	4.4 (1.6)	5.7 (1.7)	0.049	0.062	0.001 ^‡^

Mean and standard deviation (SD) are shown. Significant differences (at the same timepoint) between the control day and the alcohol day (*p* < 0.0071, after Bonferroni’s correction for multiple comparisons) are indicated by *. Significant differences (at the same timepoint) between the hangover-resistant group and the hangover-sensitive group (*p* < 0.0071, after Bonferroni’s correction for multiple comparisons) are indicated by ^‡^. Abbreviations: R = hangover-resistant group, S = hangover-sensitive group.

**Table A26:** POMS-SF—Depression.

Group	Hangover-Resistant Group			Hangover-Sensitive Group			R vs. S Group, *p*-Value	
Time	Control Day	Alcohol Day	*p*-Value	Control Day	Alcohol Day	*p*-Value	Control Day	Alcohol Day
09:30	0.43 (1.6)	1.07 (3.7)	−	0.47 (1.1)	3.27 (5.6)	0.114	0.425	0.146
10:30	0.07 (0.3)	0.93 (3.5)	−	0.40 (1.1)	3.20 (4.4)	0.095	0.561	0.093
11:30	0.0 (0.0)	0.93 (3.5)	−	0.40 (1.3)	2.07 (3.4)	0.148	0.561	0.172
12:30	0.14 (0.5)	1.14 (4.3)	−	0.73 (1.6)	2.27 (3.8)	0.527	0.377	0.172
13:30	0.14 (0.5)	1.00 (3.7)	−	0.53 (1.6)	2.20 (3.3)	0.136	0.591	0.172
14:30	0.07 (0.3)	1.00 (3.7)	−	0.73 (1.9)	1.87 (3.4)	0.391	0.533	0.290
15:30	0.14 (0.5)	1.00 (3.7)	−	0.50 (1.3)	1.27 (3.1)	0.557	0.541	0.425

Mean and standard deviation (SD) are shown. No significant differences (at the same timepoint) between the control day and the alcohol day (*p* < 0.0071, after Bonferroni’s correction for multiple comparisons) were found. For the hangover-resistant group, the main effect of control vs. alcohol day was not significant (*p* = 0.996). Therefore, no paired comparisons were conducted (indicated by −). No significant differences (at the same timepoint) between the hangover-resistant group and the hangover-sensitive group (*p* < 0.0071, after Bonferroni’s correction for multiple comparisons) were found. Abbreviations: R = hangover-resistant group, S = hangover-sensitive group.

**Table A27:** POMS-SF—Anger.

Group	Hangover-Resistant Group			Hangover-Sensitive Group			R vs. S Group, *p*-Value	
Time	Control Day	Alcohol Day	*p*-Value	Control Day	Alcohol Day	*p*-Value	Control Day	Alcohol Day
09:30	0.21 (0.4)	0.64 (1.9)	−	0.53 (0.8)	3.40 (3.3)	0.012	0.477	0.008
10:30	0.14 (0.4)	0.36 (1.1)	−	0.13 (0.5)	3.07 (3.7)	0.003 *	0.780	0.020
11:30	0.07 (0.3)	0.64 (2.1)	−	0.13 (0.5)	2.53 (3.0)	0.010	1.000	0.033
12:30	0.14 (0.4)	0.50 (1.6)	−	0.60 (1.1)	1.80 (2.7)	0.278	0.354	0.077
13:30	0.29 (1.1)	0.43 (1.3)	−	0.20 (0.6)	2.00 (3.6)	0.025	0.813	0.077
14:30	0.14 (0.4)	0.57 (1.9)	−	0.27 (0.6)	1.53 (2.3)	0.078	0.780	0.146
15:30	0.29 (0.6)	0.64 (1.6)	−	0.29 (0.6)	1.60 (2.5)	0.168	1.000	0.377

Mean and standard deviation (SD) are shown. Significant differences (at the same timepoint) between the control day and the alcohol day (*p* < 0.0071, after Bonferroni’s correction for multiple comparisons) are indicated by *. For the hangover-resistant group, the main effect of control vs. alcohol day was not significant (*p* = 0.929). Therefore, no paired comparisons were conducted (indicated by −). No significant differences (at the same timepoint) between the hangover-resistant group and the hangover-sensitive group (*p* < 0.0071, after Bonferroni’s correction for multiple comparisons) were found. Abbreviations: R = hangover-resistant group, S = hangover-sensitive group.

**Table A28:** POMS-SF—Tension.

Group	Hangover-Resistant Group			Hangover-Sensitive Group			R vs. S Group, *p*-Value	
Time	Control Day	Alcohol Day	*p*-Value	Control Day	Alcohol Day	*p*-Value	Control Day	Alcohol Day
09:30	0.43 (0.8)	0.86 (2.9)	−	1.07 (0.5)	1.60 (2.5)	0.752	0.186	0.158
10:30	0.21 (0.6)	0.50 (1.9)	−	0.47 (0.7)	1.93 (2.7)	0.030	0.400	0.046
11:30	0.43 (0.8)	0.64 (2.1)	−	0.53 (0.7)	1.80 (2.3)	0.090	0.683	0.046
12:30	0.14 (0.4)	0.43 (1.6)	−	0.80 (0.9)	2.00 (2.3)	0.040	0.093	0.004 ^‡^
13:30	0.21 (0.6)	0.50 (1.9)	−	1.07 (1.4)	1.53 (2.0)	0.603	0.112	0.051
14:30	0.21 (0.6)	0.64 (2.1)	−	1.07 (1.4)	1.60 (1.7)	0.198	0.070	0.007
15:30	0.29 (0.8)	0.79 (2.7)	−	0.57 (0.9)	1.40 (1.8)	0.114	0.376	0.085

Mean and standard deviation (SD) are shown. No significant differences (at the same timepoint) between the control day and the alcohol day (*p* < 0.0071, after Bonferroni’s correction for multiple comparisons) were found. For the hangover-resistant group, the main effect of control vs. alcohol day was not significant (*p* = 0.747). Therefore, no paired comparisons were conducted (indicated by −). Significant differences (at the same timepoint) between the hangover-resistant group and the hangover-sensitive group (*p* < 0.0071, after Bonferroni’s correction for multiple comparisons) are indicated by ^‡^. Abbreviations: R = hangover-resistant group, S = hangover-sensitive group.

**Table A29:** POMS-SF—Fatigue.

Group	Hangover-Resistant Group			Hangover-Sensitive Group			R vs. S Group, *p*-Value	
Time	Control Day	Alcohol Day	*p*-Value	Control Day	Alcohol Day	*p*-Value	Control Day	Alcohol Day
09:30	1.00 (1.6)	1.86 (2.3)	−	1.73 (1.4)	10.27 (5.0)	<0.001 *	0.070	<0.001 ^‡^
10:30	0.86 (1.1)	1.71 (1.7)	−	0.87 (0.7)	9.13 (4.2)	<0.001 *	0.683	<0.001 ^‡^
11:30	0.57 (1.1)	1.14 (1.3)	−	0.67 (0.7)	8.13 (4.0)	<0.001 *	0.425	<0.001 ^‡^
12:30	0.93 (1.5)	1.57 (2.0)	−	0.93 (1.2)	8.53 (5.0)	<0.001 *	0.715	<0.001 ^‡^
13:30	0.93 (1.3)	1.21 (1.8)	−	1.40 (1.5)	7.60 (4.3)	<0.001 *	0.377	<0.001 ^‡^
14:30	1.00 (2.1)	1.07 (0.6)	−	0.93 (1.2)	6.47 (4.0)	<0.001 *	0.561	<0.001 ^‡^
15:30	1.14 (1.6)	0.93 (1.4)	−	1.86 (2.4)	6.13 (4.3)	0.021	0.401	<0.001 ^‡^

Mean and standard deviation (SD) are shown. Significant differences (at the same timepoint) between the control day and the alcohol day (*p* < 0.0071, after Bonferroni’s correction for multiple comparisons) are indicated by *. For the hangover-resistant group, the main effect of control vs. alcohol day was not significant (*p* = 0.108). Therefore, no paired comparisons were conducted (indicated by −). Significant differences (at the same timepoint) between the hangover-resistant group and the hangover-sensitive group (*p* < 0.0071, after Bonferroni’s correction for multiple comparisons) are indicated by ^‡^. Abbreviations: R = hangover-resistant group, S = hangover-sensitive group.

**Table A30:** POMS-SF—Vigor.

Group	Hangover-Resistant Group			Hangover-Sensitive Group			R vs. S Group, *p*-Value	
Time	Control Day	Alcohol Day	*p*-Value	Control Day	Alcohol Day	*p*-Value	Control Day	Alcohol Day
09:30	12.07 (2.4)	7.93 (4.7)	0.005 *	12.07 (2.6)	2.80 (2.2)	<0.001 *	0.847	<0.001 ^‡^
10:30	12.79 (2.9)	7.64 (4.9)	<0.001 *	10.73 (2.8)	2.53 (2.7)	<0.001 *	0.102	0.002 ^‡^
11:30	12.71 (3.1)	8.79 (4.9)	0.003 *	10.13 (2.1)	2.73 (2.6)	<0.001 *	0.012	<0.001 ^‡^
12:30	10.93 (4.6)	8.43 (5.7)	0.061	9.27 (3.6)	2.60 (3.1)	<0.001 *	0.451	0.001 ^‡^
13:30	11.36 (5.2)	8.57 (5.3)	0.032	8.47 (4.0)	3.67 (3.4)	0.007 *	0.134	0.008
14:30	11.29 (4.9)	9.64 (5.4)	0.136	9.47 (3.2)	4.27 (3.8)	<0.001 *	0.270	0.007 ^‡^
15:30	10.86 (4.1)	9.64 (5.6)	0.982	7.64 (3.1)	4.60 (3.5)	0.052	0.035	0.016

Mean and standard deviation (SD) are shown. Significant differences (at the same timepoint) between the control day and the alcohol day (*p* < 0.0071, after Bonferroni’s correction for multiple comparisons) are indicated by *. Significant differences (at the same timepoint) between the hangover-resistant group and the hangover-sensitive group (*p* < 0.0071, after Bonferroni’s correction for multiple comparisons) are indicated by ^‡^. Abbreviations: R = hangover-resistant group, S = hangover-sensitive group.
